# Aqueous Geochemical Controls on the Sestonic Microbial Community in Lakes Michigan and Superior

**DOI:** 10.3390/microorganisms11020504

**Published:** 2023-02-17

**Authors:** Asha Rani, Ravi Ranjan, Solidea M. C. Bonina, Mahsa Izadmehr, John P. Giesy, An Li, Neil C. Sturchio, Karl J. Rockne

**Affiliations:** 1 Department of Civil, Materials, and Environmental Engineering, University of Illinois at Chicago, Chicago, IL 60607, USA; 2 Genomics Resource Laboratory, Institute for Applied Life Sciences, University of Massachusetts, Amherst, MA 01003, USA; 3 Department of Veterinary Biomedical Sciences and Toxicology Centre, University of Saskatchewan, Saskatoon, SK S7N 5C5, Canada; 4 Department of Environmental Science, Baylor University, One Bear Place 97266, Waco, TX 76706, USA; 5 Department of Zoology and Center for Integrative Toxicology, Michigan State University, East Lansing, MI 48824, USA; 6 School of Public Health, University of Illinois at Chicago, Chicago, IL 60612, USA; 7 Department of Earth Sciences, University of Delaware, Newark, DE 19716, USA

**Keywords:** Laurentian Great Lakes, Lake Michigan, Lake Superior, microbiome, 16S rRNA, microbial diversity, archaea

## Abstract

Despite being the largest freshwater lake system in the world, relatively little is known about the sestonic microbial community structure in the Laurentian Great Lakes. The goal of this research was to better understand this ecosystem using high-throughput sequencing of microbial communities as a function of water depth at six locations in the westernmost Great Lakes of Superior and Michigan. The water column was characterized by gradients in temperature, dissolved oxygen (DO), pH, and other physicochemical parameters with depth. Mean nitrate concentrations were 32 μmol/L, with only slight variation within and between the lakes, and with depth. Mean available phosphorus was 0.07 μmol/L, resulting in relatively large N:P ratios (97:1) indicative of P limitation. Abundances of the phyla Actinobacteria, Bacteroidetes, Cyanobacteria, Thaumarchaeota, and Verrucomicrobia differed significantly among the Lakes. *Candidatus Nitrosopumilus* was present in greater abundance in Lake Superior compared to Lake Michigan, suggesting the importance of ammonia-oxidating archaea in water column N cycling in Lake Superior. The Shannon diversity index was negatively correlated with pH, temperature, and salinity, and positively correlated with DO, latitude, and N_2_ saturation. Results of this study suggest that DO, pH, temperature, and salinity were major drivers shaping the community composition in the Great Lakes.

## 1. Introduction

The Laurentian Great Lakes, hereafter referred to as the Great Lakes, are a series of interconnected freshwater lakes located in North America. The Great Lakes form the largest group of freshwater lakes on Earth, containing 21% of the volume of the world’s surface fresh water [1]. These lakes possess sea-like characteristics, such as rolling waves, sustained winds, strong currents, great depths, and distant horizons, and have been referred to as inland seas [2]. As a source of water, transportation, food, and recreation, the Great Lakes have had a major influence on the history and development of the United States and Canada [3]. Interestingly, Lake Superior and, to a lesser extent, Lake Michigan, are known to have small concentrations of total phosphorus (TP), typically below 0.01 µmol/L, with comparatively greater concentrations of nitrate and a resultant large N:P ratio [4]. This is particularly true when compared to the 16:1 N:P “Redfield ratio” of typical marine and oligotrophic lacustrine ecosystems.

Microbes inhabit all freshwater habitats and are one of the major drivers regulating biogeochemical cycles of lakes. However, geologically rapid changes in ecosystem state caused by nutrient loading through municipal waste discharges, air- and waterborne pollution, as well as changes in climate and other anthropogenic activities have raised serious concerns about the health of the Great Lakes [1]. This has resulted in massive changes at all biotic levels, resulting in deterioration of water quality and the diversity of macro flora, including aquatic animals, plants, and microflora [1,5]. As one example, anthropogenic activities have resulted in increased surface temperatures, which can lead to unfavorable shifts in the structure of the microbial community, including nuisance cyanobacteria species, such as *Microcystis* spp. and *Cylindrospermopsis* spp. Investigating and identifying the composition of the microbial communities of these lakes at different spatial and temporal scales will enable better understanding of the microbial ecology and help determine how significant microbial interactions respond to and influence the overall status and trends in conditions of the Great Lakes. 

It has been shown that lakes within the same geographic region can vary largely in spatial and temporal conditions [6]. These differences are caused by differences in environmental exchanges including runoff, outflow, and atmospheric loading, as well as differences in physical and geochemical states that can affect structures and functions of microbial communities and the biogeochemical processes that they mediate [7]. Microbial communities regulate essential processes, such as nutrient cycling, which controls overall ecological state through interactions between primary production and organic matter processing [8]. Abundance of individual taxa relative to the numerically dominant microbial community members can be influenced by changes in physicochemical properties [9,10]. Thus, it is particularly important to understand microbial communities throughout the water column, particularly in deep lakes where gradients prevent continuous mixing. 

In holomictic lakes, mixing of the water column, turnover, and subsequent return to stratified conditions occurs seasonally. This phenomenon can influence structures of microbial communities as a consequence of shifting environmental conditions [11,12,13]. Thermal stratification of water at different temperatures and densities results in a hypolimnion that is colder with lesser concentrations of dissolved oxygen (DO) and pH relative to the epilimnion, and inorganic nutrients typically accumulate in the hypolimnion [14,15]. Stratification is a major disturbance that will likely affect structures of microbial communities as the lake gradually stratifies post-mixing. Results of studies have suggested that microbial communities respond to turnover with various degrees of resilience, and some communities may remain unaffected by the disturbance [16,17]. 

Mixing of lakes can also transport dissolved organic carbon (DOC) throughout the water column [18,19,20], which is important because DOC plays a key role in shaping compositions of sestonic microbial communities [21,22]. Following stratification, accumulation and remineralization of organic matter in the hypolimnion are important biogeochemical processes [23,24]. The phyla Actinobacteria, Proteobacteria, and Bacteroidetes are globally predominant in freshwater systems [25]. However, other phyla, such as Chloroflexi, Thaumarchaeota Marine Group I, and members of Planctomycetes also dominate the oxygenated hypolimnion [26,27,28,29,30]. Although these constituents are important components of the lacustrine microbial food web, it is unclear how abundant they are, and their ecological importance to functional metabolic diversity of the freshwater ecosystem is not well described. 

The main goal of this study was to examine the bacterial community diversity as a function of depth in the water column at several locations roughly along a north–south transect in Lake Michigan (LM) and Lake Superior (LS). Lakes Michigan and Superior span nearly 600 km east to west and over 800 km north to south. Based on nutrient status, Lakes Michigan and Superior are generally classified as oligotrophic [4,31,32,33]. Both lakes stratify seasonally, with a substantial hypolimnion developed below the thermocline. However, these lakes have unique and contrasting physicochemical properties. The objective of this study was to characterize the microbial community structure and diversity within each lake and across geographically distant sampling locations to analyze temporal and spatial variability. 

## 2. Materials and Methods

### 2.1. Sampling 

**Figure 1:**
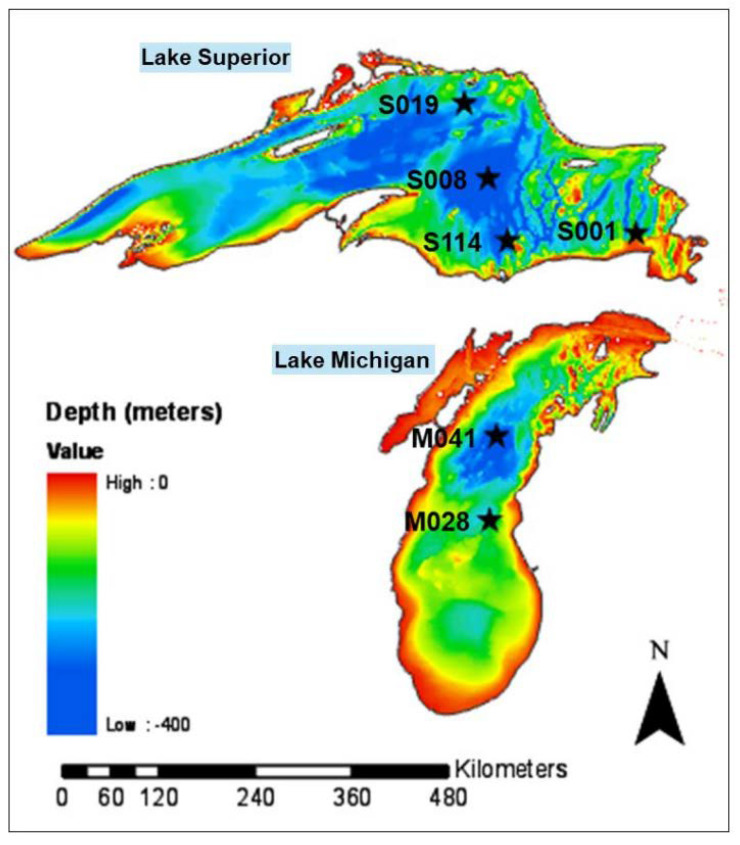
Sampling locations in Lake Michigan and Lake Superior. Sample collection locations in Lake Michigan (M028 and M041) and Lake Superior (S001, S008, S019, and S114) are shown with a star symbol. Geographical locations of the sampling sites are provided in Table S1.

Samples of water were collected from LM and LS in September 2010 and May 2011 onboard the US Environmental Protection Agency (USEPA) *R/V Lake Guardian*. Two sampling locations from LM (M028 and M041) and four sampling locations from LS (S001, S008, S019, and S114) were included in this study (Figure 1, Table S1). This sample collection trip was ad hoc for this research and was not part of the seasonal cruises. At all locations, samples of water were obtained at depths of 5, 10, 20 and 50 m, and then every 50 m to the profundal zone with 2 additional samples at 2 and 10 m from the sediment-water interface. In total, 16 samples of water were collected across 2 sampling locations of LM and 33 samples of water across the 4 locations in LS. The sampling locations have been sampled in several previous studies as points of comparison [34,35,36,37,38,39,40]. Sampling details are provided in supplementary data.

### 2.2. Extraction, PCR Amplification, and Sequencing of DNA

DNA was extracted using the PowerSoil DNA isolation kit following the manufacturer’s protocol (Qiagen, Inc, Germantown, MD, USA). A standard DNA extraction protocol from the Earth Microbiome Project (EMP) was used (http://www.earthmicrobiome.org/protocols-and-standards/dna-extraction-protocol. The detailed methods are provided in supplementary data. 

### 2.3. Bioinformatics and Statistical Analysis

Reads were quality filtered and taxonomic annotations were obtained using the automated annotation pipeline at MG-RAST web server [http://metagenomics.anl.gov/]. The best hit classification was used with a maximum *e*-value 1 × 10^−5^ cutoff and a minimum 97% identity cutoff using the Greengenes database [41,42]. The quality-filtered OTU table was compared at the phylum and genus level, with taxa comprising ≥1% of the reads defined as abundant. Relative abundance for each taxon was normalized by the percentages of respective reads over the total assigned reads of the microbiome. Data corresponding to taxonomical distributions and sample comparisons were analyzed with the STAMP ver. 2.0 software [43]. The detailed methods are provided in supplementary data. LDA-based LEfSe approach (linear discriminant analysis effect size) was used as the statistical biomarker tool to identify preferentially abundant taxa across LM and LS as well as among various sampling locations at phylum and genus level [44]. LEfSe analysis was run using Calypso web server [45] with bootstrap iterations of 30 and minimum effect size of 3.0. The detailed methods are provided in supplementary data.

### 2.4. Molecular Phylogenetic Analysis for Thaumarchaeota

A 16S rRNA gene-based phylogenetic tree of closest known sequences within the *Thaumarchaeota* from Lakes Michigan and Superior was constructed. The tree was based on the aligned representative sequences for 14 OTUs from LM and 32 OTUs from LS identified in this study. Representative sequences for Archaea were sequence matched using the National Center for Biotechnology Information (NCBI) genome browser for each OTU. All nucleotide sequences were aligned using ClustalW and a phylogenetic tree was constructed using MEGA version 4.0 [46]. The method details are provided in the supplementary data. 

## 3. Results and Discussion

### 3.1. Spatial Variation in Physicochemical Parameters

**Figure 2:**
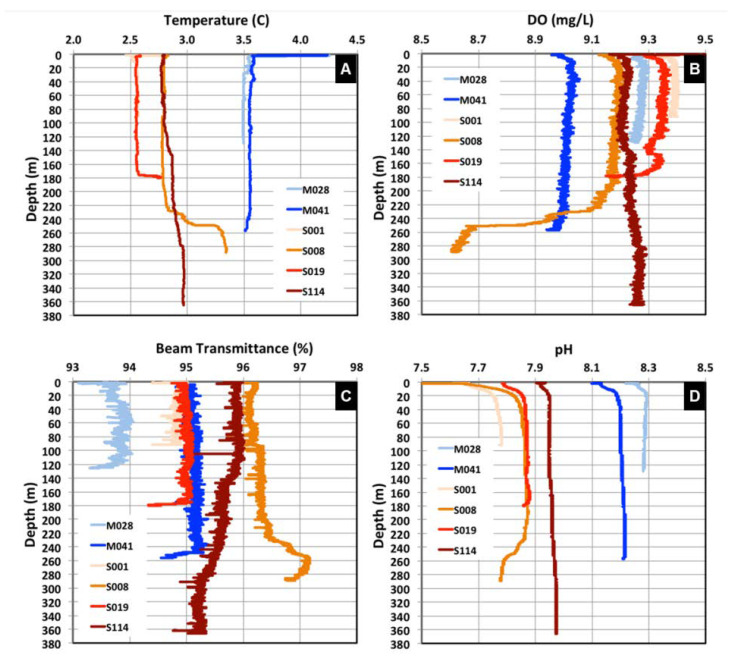
Water column profiles of physical and chemical parameters in Lakes Michigan and Superior. Shown are (**A**) temperature, (**B**) dissolved oxygen (DO), (**C**) beam transmittance, and (**D**) pH as a function of depth.

**Figure 3:**
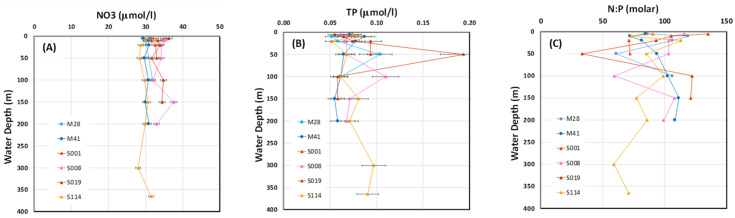
Nutrients in LM and LS sample locations. Shown are (**A**) nitrate (NO3), (**B**) total phosphorus (TP), and (**C**) molar NO3- N:TP ratio in filtered LM and LS samples. Note that not all samples were available for analysis. Error bars on x axis data represent the 95% uncertainty (U95), which was 2.3% and 12.9% for nitrate and TP, respectively.

Great differences in aqueous geochemical parameters were observed between Lakes Michigan and Superior (Figure 2 and Figure S1, Table S1). The LM water column had statistically significantly (*p* < 0.0001) greater pH, temperature, and salinity compared to LS (Figure S1). In general, the aqueous geochemical parameters were more similar between LM sampling locations M028 and M041, while LS aqueous geochemical parameters varied to a greater extent among sampling locations and depths. Greater N_2_ saturation and relatively comparable concentrations of DO were observed among sampling locations in LS compared to those among LM locations. In sampling location S008, samples at depths 2 m off bottom and 10 m off bottom revealed a distinct pattern for temperature, pH, DO, and N_2_ saturation compared to other depths, while no significant differences were detected in salinity among these depths. At all locations, profiles of temperature, DO, beam transmission, and pH clearly demonstrated stratification (Figure 2). The bottom water in LM had greater pH, lower temperature, beam transmission, and DO compared to the surface water. In contrast, the bottom water in LS had greater pH, concentrations of DO, and temperature, with lesser beam transmission than the surface water. Comparing within LS, site S008 had lower concentrations of DO and greater beam transmission compared to other locations within the lake (Figure 2, Table S1). A Jaccard distance matrix shows the heatmap for variability among aqueous chemical parameters across sampling locations and depths (Figure S2). In contrast to physicochemical parameters, there was only a small variation in nutrient concentrations between and among lakes and with depth (Figure 3A,B). The mean concentration of NO_3_^−^ was 31 ± 1 µmol/L in LM and 33 ± 2 µmol/L in LS, with no significant difference (95% CI) observed in benthic samples (2 and 10 m off the bottom) compared to those in the water column (Figure 3A). The only statistically significant difference in NO_3_^-^ (*p* < 0.001) was observed between site S114 and locations S008 and S019. There were no statistically significant differences in dissolved TP between and among lakes (0.066 ± 0.014 µmol/L and 0.073 ± 0.029 µmol/L in LM and LS, respectively), except for the peak in TP at 50 m depth at site S019 (Figure 3B). These relatively large N and relatively small TP concentrations resulted in large N:P ratios at all locations, averaging 97:1 on a molar basis (Figure 3C), a value greater than the 16:1 Redfield ratio typically observed in marine and oligotrophic lacustrine locations, suggestive of P limitations. Both the NO_3_^−^ and TP concentrations are consistent with published results compiled for LS [4], with averages of 24–27 µmol/L and 0.036 µmol/L for NO_3_^−^ and dissolved TP, respectively. 

### 3.2. Microbial Community Structure

**Figure 4:**
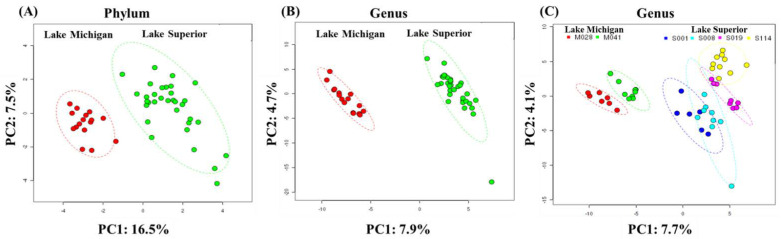
Principal coordinate analysis of the bacterial diversity at phylum level (**A**) and genus level (**B**) in Lakes Michigan and Superior. Also shown are (**C**) genus level diversity in different sampling locations in Lakes Michigan and Superior. Each dot in the graph represents a sample and color circles indicate various sampling locations. Red circles represent Lake Michigan and light green represent Lake Superior samples. The Lake Michigan and Lake Superior groups are well separated and could be differentiated readily using variation of the first two components (PC1 and PC2) of the PCA plot and noted in both axis labels. Similar direction and magnitude of clustering indicate a large positive association among the lake types. A 95% confidence interval was used as the threshold to identify potential outliers.

Sample description and sequence details are provided in Table S2. The microbiome data for each sample was ordinated by applying principal coordinate analysis (PCoA) techniques (Figure 4). PCoA identified distinct clusters of LM and LS samples at both the phylum (Figure 4A) and genus (Figure 4B) levels. PC1 and PC2 explained 16.5% and 7.9% of the total variance among the samples for phylum and genus level, respectively. A 95% confidence interval threshold showed a group separation of the LM and LS samples both at phylum and genus level. Separation of the LM and LS microbiomes was determined by use of Ward’s hierarchical clustering method using Pearson’s correlation similarity (Figure S3). The dendrogram confirmed segregation of the LM and LS samples similar to the PCoA analysis. LM samples were similar to one another, as were LS samples. To determine the microbial diversity structure of different sampling locations, the microbial taxa in the lakes were analyzed by use of PCoA analysis (Figure 4C). Together, these analyses indicate that the LM and LS have markedly different microbial community compositions.

### 3.3. Microbial Taxa and Diversity Indices between Lakes

**Figure 5:**
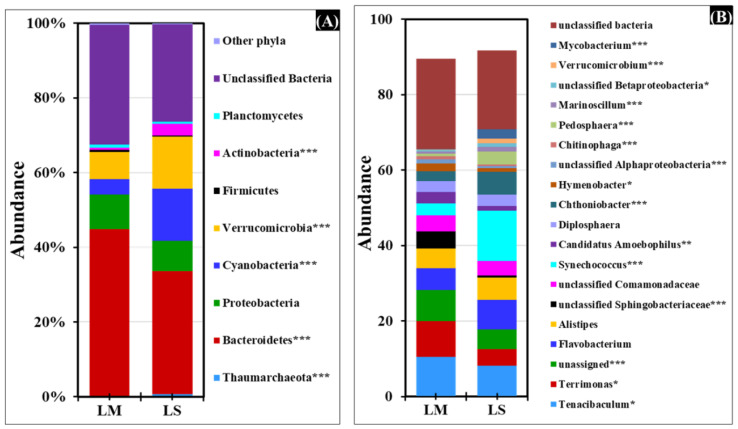
Stacked bar chart representing the relative abundance of different (**A**) phyla and (**B**) genera/families across Lakes Michigan and Superior. The top 20 taxa, by abundance, are shown for clarity. Significant differences among the lakes were computed using Mann–Whitney *U* test. * *p* < 0.05, ** *p* < 0.001, and *** *p* <0.0001. The significantly different phyla/genera/families among the lakes are shown in the figure description.

Microbiomes in both Lakes Michigan and Superior were dominated by two major bacterial phyla: Bacteroidetes (32–44%) and unclassified bacteria (26–32%). Proteobacteria were similar in abundance in both Lakes (8–9%) whereas Cyanobacteria and Verrucomicrobia were more abundant in LS (13–14%) compared to LM (4–7%) (Figure 5A, Table S3). The archaeal phyla *Thaumarchaeota* was detected in both LM and LS (0.2–0.6%) in low abundance. Phylum Actinobacteria was much greater in abundance in LS (3.1%) compared to LM (0.7%). Overall, the phyla Actinobacteria, Bacteroidetes, Cyanobacteria, Thaumarchaeota, and Verrucomicrobia were statistically significantly different in microbial abundance between the lakes. To focus on more specific classification between samples, data were analyzed at the genus level (Figure 5B, Table S4). A total of 144 genus level OTUs were identified in LM and 118 in LS. Of these 262 OTUs, 64% (102) were shared, 26% (42) were exclusive to LM, and 16% (10) were identified in LS only (Figure S4A–C). The top 19 genera/families, comprising >1% abundance between lakes, are shown in Figure 5B. The Shannon diversity index and species evenness values were greatest for LS compared to LM (Figure S5). The results of a one-way ANOVA analysis indicate that significantly (*p* < 0.0001) greater Shannon diversity was observed in near bottom-water depths compared to surface-water column sample locations (Figure 2 and Figure S5). Some studies have suggested that anoxic hypolimnion microbial communities are more diverse (α-diversity) than in the epilimnion [47,48,49], while others have found no significant difference in diversity [30].

### 3.4. Microbial Community Structure in Different Sampling Locations

**Figure 6:**
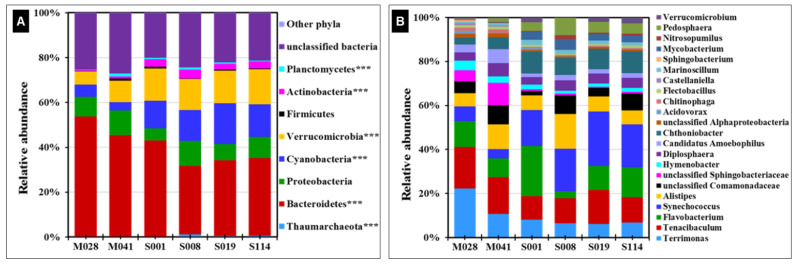
Stacked bar chart representing the relative abundance of different (**A**) phyla and (**B**) genera/families across various sampling locations in Lakes Michigan and Superior. The top 22 taxa, by abundance, are shown for clarity. Significant differences among the lake types were computed using one-way ANOVA with Tukey’s post hoc test for multiple comparisons, *** *p* < 0.0001. Significantly different phyla/genera/families among the lakes are shown in the figure description.

In all the sampling locations, 20–26% of the microbial community was identified as unclassified bacteria (Figure 6A, Table S5). Among the identified bacterial phyla, Bacteroidetes and Cyanobacteria were more abundant (54% vs. 45%, and 6% vs. 4%, respectively; *p* < 0.001) at sampling location M028 compared to M041 (Figure 6A, Table S5). The phylum Verrucomicrobia was greater in abundance (9.6%) in sampling location M041 compared to M028 (5.8%) in LM. There were no significant differences in the phyla Actinobacteria, Proteobacteria, and unclassified bacteria among these two sampling locations in LM. The phylum Bacteroidetes (42.6%) was detected in great abundance at S001 compared to sampling locations S008, S019, and S114. In addition, the phyla Cyanobacteria (12–18%), Verrucomicrobia (14–16%), and Actinobacteria (2.5–4%) were significantly greater across all sampling locations in LS compared to LM (Figure 6A, Table S5). There was no significant difference among the phyla Proteobacteria and unclassified bacteria in all sampling locations in LS. Of particular interest, the only Archaeal phylum identified in all locations was *Thaumarchaeota*, which varied on average between 0.2% total abundance in LM and 0.6% in LS. The greatest abundance, of 1.3%, was detected at LS site S008. A genus-level analysis provides the most in-depth details on the microbiome composition between and among sample locations (Figure 6B, Table S6). Each sampling location revealed a characteristic site-specific microbial profile that was readily distinguishable from other locations (Figure S6). The genera *Terrimonas* and *Synechococcus* were abundant at specific depths across lakes, while the genera *Alistipes* and *Flavobacterium* were predominant only at sampling locations S008 and S001, respectively (Figure S14). Microbial diversity at phylum level across different depths provided further detailed description for the microbial diversity between lakes (Figures S13 and S7A–H). At M041, Bacteroidetes abundance was lesser than that at M028 (Figure S7F). As expected, photosynthetic Cyanobacteria were almost absent (<1% abundance) in deep locations at 2 and 10 m off bottom compared to near-surface samples at most sites.

### 3.5. Distribution of Thaumarchaeota

**Figure 7:**
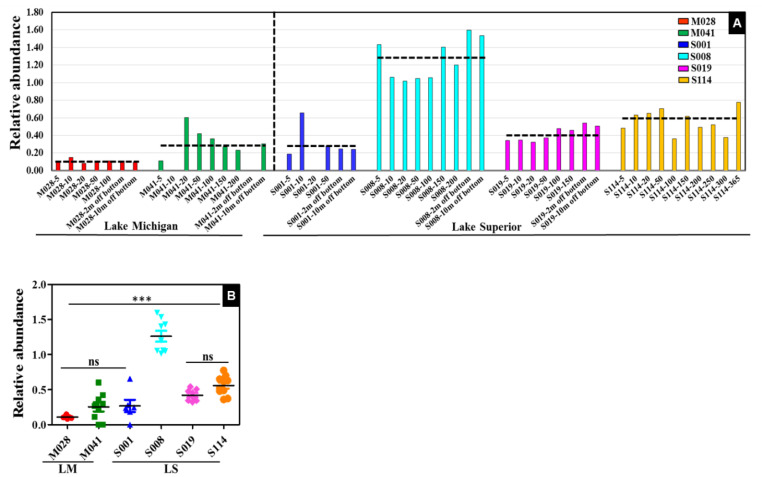
Distribution of Thaumarchaeota in the western Great Lakes. Shown are (**A**) relative abundance of phylum Thaumarchaeota at different depths in Lake Michigan and Lake Superior sampling locations, with relative abundance and sample name followed by depth shown in the x and y axes, respectively. Horizontal dashed line represents the mean abundance within each group. Lakes are separated by vertical black dashed lines. Samples are color coded to reflect sampling location in both the lakes. (**B**) The significant differences among the sampling locations for phylum Thaumarchaeota were computed using one-way ANOVA with Tukey’s post hoc test for multiple comparisons. *** *p* < 0.0001.

Relative abundances of phylum *Thaumarchaeota* at various depths in LM and LS sampling locations were significantly different (*p* < 0.0001) (Figure 7A,B). LS archaeal sequences were more closely related to each other than to LM sequences (Figure S8). The greatest abundance of *Thaumarchaeota* (1.4–1.6%) was detected in sampling location S008 in samples from near the bottom (50–200 m, 2–10 m off bottom) compared to surface samples (1–1.4%) at 5 to 20 m depths. Sampling location S008 had the greatest abundance of *Thaumarchaeota* compared to all other locations. At sampling location S114, *Thaumarchaeota* abundance varied from 0.4–0.8%, and was greatest (0.8%) at the deepest site (365 m) sampled in this study (Figure 7A,B). Prior to the discovery of ammonia-oxidizing archaea (AOA, phylum *Thaumarchaeota*), oxidation of ammonia was thought to be limited to certain lineages of Proteobacteria [50]. This discovery has dramatically increased our knowledge of microbial nitrogen cycling in freshwater ecosystems [51,52,53,54,55,56,57,58]. The majority of AOA present in freshwater ecosystems belong to a specific subgroup referred to as Marine Group I. Oxidation of ammonia produces protons that can create lower pH microenvironments and potentially facilitate growth of AOA in the natural environments. Additionally, AOA can also adapt to lower pH environments that might promote growth of AOA [59]. The results from this study indicate that Thaumarchaeota are present throughout the water column in LS at all sampling locations, in contrast to LM. Thaumarchaeota were most abundant at location S008 and primarily at depths of 50 m and below from the surface. These sample depths and locations also have lower temperature and pH compared to surface water, consistent with the niche partitioning of AOA abundance.

### 3.6. Biomarker Signature Analysis (LEfSe)

LEfSe analysis can be used to analyze bacterial community data at any taxonomy level, and in the present study, we conducted LEfSe analysis for each sampling location at both the phylum level (Figure S9A) and genus level for each lake (Figures S9B and S10A). A total of 6 bacterial phyla were distinct to at least 1 sampling location using the criterion of logarithmic LDA score > 3 (Figure S9A). Notably, LDA score plots only show taxa with LDA values > 3 for clarity. At the genus level, the top 24 enriched genera in different sampling locations across lakes are shown (Figure S9B). Comparisons of biomarker genera across the lakes reflected similar results to those seen in the comparison of sampling locations (Figure S10A). Using the same LDA > 3 criterion, a total of 26 characteristic biomarker taxa were identified for LM and LS (Figure S10A). Based on the Log10 odds ratio, biomarker taxa were identified; they can be used to segregate microbiomes by lake and sampling location. A detailed description of 19 significantly different (*p* < 0.0001) taxa identified are reported (Figure S10B).

### 3.7. Environmental Variation and Community Structure

**Figure 8:**
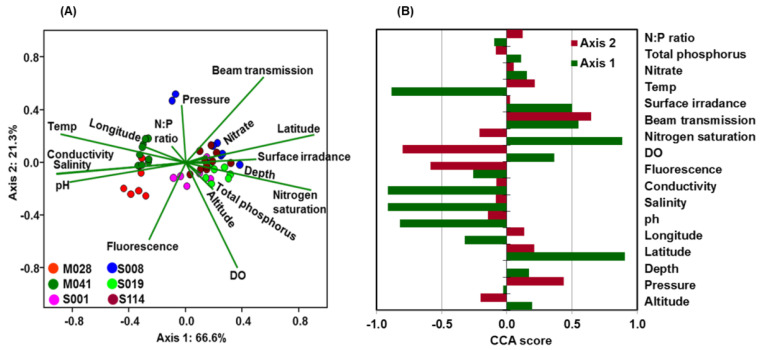
Canonical correspondence analysis (CCA) of (**A**) the environmental variables and microbial community at various depths across the lakes. Also shown is (**B**) a biplot of loading scores of environmental variables on each axis. Lines indicate direction and magnitude of measurable variables associated with community structure (loadings on each principal component). The percentage of total variance explained by each axis is noted in both the axis labels. Each symbol is color coded to represent lake or sampling locations and is provided in the figure description. Each color point represents a different bacterial community structure from each lake sample, and similar direction and magnitude of lines indicate a large positive association.

**Figure 9:**
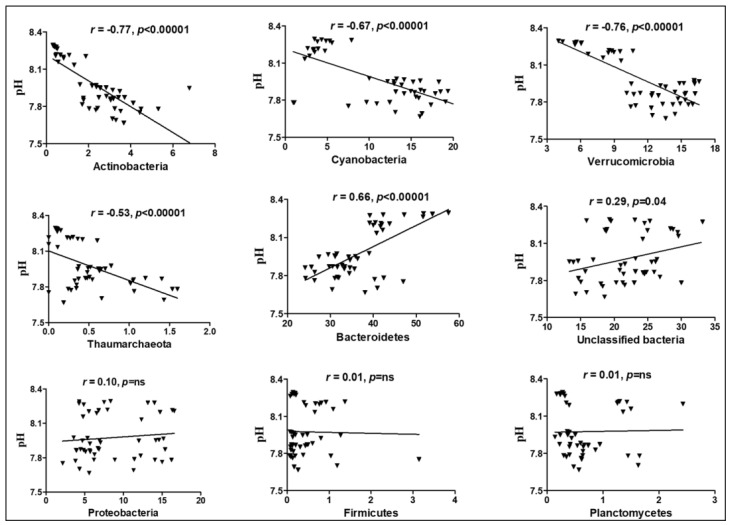
Linear regression analysis between microbial diversity at phylum level and environmental variable pH. The relative abundance and pH values are provided in x and y axes, respectively. Correlation coefficient and *p*-values are provided in the figure description.

**Figure 10:**
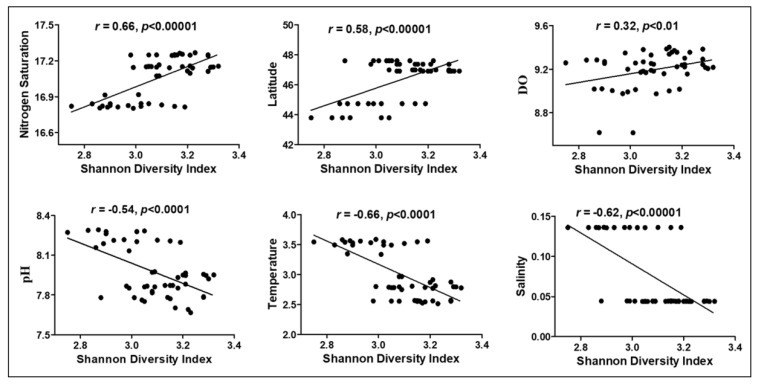
Linear regression analysis between Shannon diversity index and environmental variables. The Shannon diversity index and environmental variable values are given in the x and y axes, respectively. Correlation coefficient and *p*-values are provided in the figure description.

ANOSIM between lakes and among sampling locations identified key differences in structures of microbial communities (Figure S11A,B; ANOSIM R = 0.72, *p* < 0.0001). Significant correlations (*p* < 0.001) were found between DO, pH, temperature, and other variables and microbial beta diversity. Multivariate CCA was used to analyze variation in community structure with DO, N_2_ saturation, pH, conductivity, salinity, temperature, pressure, latitude, longitude, and depth. Collectively, these data explained 66.6% and 21.3% of the variation in community structure by the PC1 and PC2 principal components, respectively (*p* < 0.05) (Figure 8A,B). This proportion is relatively high compared to those reported in other studies of freshwater lakes [60,61]. Freshwater aquatic microbiomes are greatly influenced and significantly correlated with DO [62], and this was observed in this study since concentrations of DO were significantly (*p* < 0.001) related to compositions of microbial communities (Figure 8A, Table S7). Multiple taxonomic groups were statistically significantly correlated with environmental variables (Table S7). Specifically, members of the phylum Thaumarchaeota were positively correlated with latitude, beam transmission, and surface irradiance, and negatively correlated with pH, salinity, conductivity, and fluorescence. Proteobacteria were negatively correlated with DO while Bacteroidetes were positively correlated with pH, salinity, conductivity, and temperature, and negatively related to latitude, N_2_ saturation, beam transmission, and surface irradiance. Members of Actinobacteria, Cyanobacteria, and Verrucomicrobia were negatively correlated to pH, salinity, conductivity, and temperature, and positively correlated to latitude and N_2_ saturation. The photosynthetic Cyanobacteria were positively correlated with DO, while Planctomycetes were negatively correlated with DO. Unclassified bacteria were positively correlated with salinity, conductivity, and temperature, and negatively correlated with N_2_ saturation. Other OTUs related to unclassified *Sphingobacteriaceae* (Chitinophaga) contain aerobic microbial taxa [63] and are common in the Great Lakes [64]. Members of the phyla Proteobacteria and Planctomycetes were inversely correlated to DO. While correlations with pH suggest that preferred environmental pH can differ for individual genus level OTU, the data are too few to make generalizations across most taxonomic groups. However, there were groups of bacteria that were negatively correlated with pH, including Actinobacteria, Cyanobacteria, Verrucomicrobia, and Thaumarchaeota (Figure 9), while Bacteroidetes and unclassified bacteria were positively correlated with pH (Figure 10). Many others, including Proteobacteria, Firmicutes, and Planctomycetes, were not correlated with pH. These taxa may have been constrained by other factors (DO, temperature, or salinity), which can limit the impact of pH as a constraining variable. As pH generally decreases with increasing depth within the water column, these taxa may be more abundant near the water surface. The Shannon diversity index was also negatively correlated with pH, temperature, and salinity and positively correlated with DO, N_2_ saturation, and latitude (Figure 10).

## 4. Conclusions

The Laurentian Great Lakes are unique, interconnected aquatic ecosystems for investigating fundamental questions on microbial evolution and community structure in a natural freshwater system. Great lakes with ocean-like physical processes (strong currents and upwelling) provide a unique opportunity to study key principles that maintain these microbial communities in the freshwater ecosystems. Although they constitute a critical component of aquatic ecosystems, microbial communities of great lakes have been far less studied compared to small lake and marine ecosystems. Freshwater ecosystems along the temporal and spatial scales have the potential to uncover diversity patterns that are not apparent in the traditional studies of either bacteria or archaea in single freshwater lake systems. Our objectives were to characterize microbial community patterns across lakes, sampling depths, and locations to explore the physicochemical variables and interactions that underlie these patterns. Our study further raises new questions on how and which metabolic processes shape this wide genome diversity across different aquatic ecosystems. This study provides an extensive overview of the sestonic microbial community structure in Lakes Michigan and Superior, down to some of the deepest depths in the water column sampled. Microbial diversity analysis revealed that LM sampling locations are more similar to each other yet represent a distinct microbiome signature. Similarly, LS sampling locations represent a site-specific microbial structure and *Thaumarchaeota* was present across all depths but greatly abundant in sampling location S008. The two lakes are greatly different from each other in physicochemical variables, but surprisingly similar in nutrient concentrations of N and P. Diversity index analysis identified that the Shannon diversity index was negatively correlated with pH, temperature, and salinity, but positively correlated with DO, latitude, and N_2_ saturation. In general, LS exhibited a greater diversity index at all sampling locations and depths compared to locations in LM, with DO, pH, temperature, and salinity being the most significant drivers in shaping the microbial dynamics and composition. 

Results of this study have highlighted the findings that the microbial communities within DO, pH, and temperature-stratified lakes are greatly different from each other compared to communities within lakes that do not chemically stratify. Surprisingly, these differences do not appear to be influenced as strongly by N and P, although both nutrients are present in small concentrations relative to most mid-latitude lacustrine systems. Correlations of individual microbial phyla and OTUs with DO, pH, salinity, latitude, and temperature relate to the metabolic and functional capabilities of these microbial taxonomic groups. This also suggests that lake stratification and environmental variables unique to LM and LS may influence the adaptation and abundance of some microbial taxa more strongly than others. Such adaptation may play a role in the abundance of *Thaumarchaeota* in LS compared to LM. The strong correlation of *Thaumarchaeota* with pH in LS suggests the need to investigate the impacts of acidification. A finding that more acidic pH promotes the growth of AOA could potentially influence the ecosystem processes, such as ammonia oxidation, that are carried out by these taxa. This is further supported by phylogenetic analysis of archaeal 16S rRNA, which revealed that the archaea of LS are members of the ammonia-oxidizing Group I.1a *Thaumarchaeota* that are most closely related to *Candidatus Nitrosopumilus* sp. NM25. These AOA are distinct from the AOA in LM’s water column. Pearson correlations and canonical correspondence analysis (CCA) showed that the differences in abundance and diversity of AOA are likely related to the sampling locations and, thereby, to the different trophic states of both lakes. 

Results of this study emphasize the importance of sampling the entire water column from surface to near-bottom depths in lakes. Distinct physical and chemical attributes among lakes suggest the potential impact of lake mixing and stratification as a disturbance to microbial communities. Seasonal variations make the water column thermally stratified, and development of vertical structure in depth profiles of nutrients, and other lake physicochemical variables analyzed in this study, significantly correlate with microbial diversity at various depths and locations. Within temperate freshwater lacustrine systems, these sestonic changes could ultimately influence microbial community functional diversity and biogeochemical processes. Our findings reveal that the LM and LS microbial diversity is composed of similar microbial taxa shared across these lakes. However, they vary in abundance and community structure based on sampling depth and location in each lake. At the same time, some taxa show very strong enrichment only in certain sampling locations, such as *Synechococcus* and *Thaumarchaeota* in LS sampling stations, suggesting a critical role of selection in shaping these communities. Similarly, near the bottom, samples have lower temperature and pH compared to surface water, consistent with the niche partitioning of ammonia-oxidizing archaea abundance in LS. Of specific interest, our findings highlight a highly underexplored freshwater habitat that may foster novel metabolic interactions yet to be discovered. The temporal and spatial diversity patterns of surface-water microbial communities were relatively invariant compared to bottom-water communities that typically had great divergence among depths. This provides further evidence that lake stratification is greatly important in shaping microbial communities across both lakes. Our results provide a characteristic site-specific microbial profile that was readily distinguishable from other locations, highlighting the need to study how microbial evolution and selection shapes the microbial diversity across these extreme water ecosystems.

## Data Availability

The sequence data is available at the MG-RAST web server [41] and the accession numbers are provided in Table S2. Study description, sequencing files, and sample metadata are also available at EBI server (http://www.ebi.ac.uk/ena) with accession number ERP016492 under The Earth Microbiome Project (EMP, http://www.earthmicrobiome.org).

## Supplementary Materials

The following supporting information can be downloaded at: https://www.mdpi.com/article/10.3390/microorganisms11020504/s1.
