# Supplementary material for: Aqueous Geochemical Controls on the Sestonic Microbial Community in Lakes Michigan and Superior
Source: Microorganisms. 2023 Feb 17;11(2):504. doi: 10.3390/microorganisms11020504 (PMC9963676; doi:10.3390/microorganisms11020504)
Supplement: Supplementary file 1 [file microorganisms-11-00504-s001.zip › microorganisms-2128228 -S1.pdf]

Table S1. Environmental data from the sampling sites for Lake Michigan and Lake Superior.

Sampling station	Depth (m)	Latitude	Longitude	Altitude	Pressure	pH	Dissolved oxygen (ml/L)	Salinity (PSU)	Temperature (C)	Conductivity (mS/cm)	Nitrogen saturation (ml/L)	Beam transmission (chelsea/Seatech) %	SPAR/Surface irradance	Fluorescence	Nitrate (µmol/L)	Total phosphorus (µmol/L)	N:P molar)
M028-5	5	43.80	-86.80	98.61	4.99	8.26	9.27	0.14	3.53	0.17	16.83	93.74	1380.00	4.07	31.29	0.07	86.09
M028-10	10	43.80	-86.80	98.61	9.82	8.27	9.26	0.14	3.54	0.17	16.82	93.74	1390.00	6.37	30.07	0.05	118.95
M028-20	20	43.80	-86.80	98.58	19.84	8.29	9.29	0.14	3.54	0.17	16.82	93.69	1380.00	8.94	30.21	0.06	106.23
M028-50	50	43.80	-86.80	84.86	49.05	8.29	9.28	0.14	3.49	0.17	16.84	93.97	1380.00	8.24	30.86	0.10	61.03
M028-100	100	43.80	-86.80	32.38	98.08	8.28	9.27	0.14	3.49	0.17	16.84	93.94	1360.00	8.83	31.86	0.06	106.11
M028-2m off bottom	2	43.80	-86.80	4.20	124.61	8.28	9.25	0.14	3.49	0.17	16.84	93.46	1360.00	8.39	30.21	0.05	119.51
M028-10m off bottom	10	43.80	-86.80	12.67	116.70	8.28	9.26	0.14	3.49	0.17	16.84	93.69	1360.00	8.90	29.64	0.05	110.35
M041-5	5	44.74	-86.72	98.61	4.98	8.13	8.99	0.14	3.59	0.17	16.80	95.02	1.97	6.89	29.43	0.07	84.66
M041-10	10	44.74	-86.72	98.61	9.89	8.16	9.02	0.14	3.58	0.17	16.81	95.05	1.97	6.26	30.79	0.09	72.16
M041-20	20	44.74	-86.72	98.61	19.66	8.19	9.02	0.14	3.56	0.17	16.81	95.17	1.97	6.52	30.93	0.08	81.56
M041-50	50	44.74	-86.72	92.70	49.03	8.20	9.02	0.14	3.56	0.17	16.81	95.12	1.97	6.70	29.64	0.06	93.80
M041-100	100	44.74	-86.72	98.61	98.13	8.20	9.01	0.14	3.55	0.17	16.82	95.22	1.97	6.26	30.79	0.06	102.54
M041-150	150	44.74	-86.72	98.61	147.16	8.21	9.00	0.14	3.55	0.17	16.82	95.10	1.97	6.01	29.93	0.05	111.42
M041-200	200	44.74	-86.72	98.61	196.17	8.21	9.00	0.14	3.56	0.17	16.81	95.20	1.97	6.85	30.86	0.06	108.49
M041-2m off bottom	2	44.74	-86.72	4.71	249.10	8.21	8.97	0.14	3.52	0.17	16.83	95.15	1.97	6.08	33.07	0.06	104.65
M041-10m off bottom	10	44.74	-86.72	8.99	245.22	8.22	8.98	0.14	3.53	0.17	16.83	95.22	1.97	5.82	31.43	0.06	104.69
S001-5	5	46.99	-85.16	92.77	4.94	7.67	9.36	0.04	2.51	0.06	17.27	94.92	19.70	8.13	31.50	0.07	90.62
S001-10	10	46.99	-85.16	87.57	9.82	7.70	9.37	0.04	2.53	0.06	17.26	94.92	19.70	8.21	31.57		
S001-20	20	46.99	-85.16	77.07	19.61	7.75	9.38	0.04	2.56	0.06	17.25	94.84	19.70	7.84	32.64	0.09	71.24
S001-50	50	46.99	-85.16	45.64	49.04	7.77	9.40	0.04	2.57	0.06	17.25	94.58	21.70	7.40	33.00	0.09	72.02

S001-2m off bottom	2	46.99	-85.16	4.74	87.31	7.78	9.38	0.04	2.57	0.06	17.24	94.87	21.70	7.47	33.14	0.00	0.00
S001-10m off bottom	10	46.99	-85.16	14.09	78.47	7.78	9.39	0.04	2.57	0.06	17.24	94.87	21.70	7.58	34.00	0.06	107.59
S008-5	5	47.61	-86.82	98.61	4.91	7.69	9.16	0.04	2.81	0.06	17.14	96.25	1870.00	2.42	34.79	0.06	115.87
S008-10	10	47.61	-86.82	98.61	9.85	7.76	9.17	0.04	2.79	0.06	17.15	96.22	1860.00	4.10	34.93	0.07	105.26
S008-20	20	47.61	-86.82	98.61	19.62	7.83	9.19	0.04	2.79	0.06	17.15	96.17	1830.00	5.60	34.21	0.07	103.11
S008-50	50	47.61	-86.82	71.58	49.07	7.85	9.20	0.04	2.80	0.06	17.14	96.12	1790.00	5.97	34.21	0.07	103.11
S008-100	100	47.61	-86.82	93.46	98.10	7.86	9.18	0.04	2.78	0.06	17.15	96.33	1500.00	6.04	31.93	0.11	59.43
S008-150	150	47.61	-86.82	98.61	147.18	7.87	9.17	0.04	2.78	0.06	17.15	96.33	1640.00	5.90	37.57	0.07	108.08
S008-200	200	47.61	-86.82	96.43	196.19	7.87	9.16	0.04	2.81	0.06	17.14	96.40	1660.00	5.53	32.93	0.07	99.24
S008-2m off bottom	2	47.61	-86.82	4.22	281.51	7.78	8.62	0.04	3.35	0.06	16.91	96.87	1640.00	0.77	33.50	0.06	106.01
S008-10m off bottom	10	47.61	-86.82	12.31	273.70	7.78	8.62	0.04	3.34	0.06	16.92	97.02	1660.00	0.81	32.21	0.06	101.94
S019-5	5	47.37	-90.85	98.61	4.99	7.79	9.29	0.04	2.56	0.06	17.25	94.97	1600.00	2.42	36.29	0.05	135.08
S019-10	10	47.37	-90.85	98.61	9.82	7.82	9.33	0.04	2.56	0.06	17.25	94.97	1600.00	3.88	33.29	0.06	105.33
S019-20	20	47.37	-90.85	98.61	19.67	7.85	9.36	0.04	2.54	0.06	17.25	94.94	1600.00	6.63	33.93	0.07	93.36
S019-50	50	47.37	-90.85	98.58	49.05	7.87	9.35	0.04	2.56	0.06	17.25	95.07	1590.00	6.63	31.64	0.19	33.38
S019-100	100	47.37	-90.85	86.37	98.08	7.87	9.36	0.04	2.56	0.06	17.25	95.02	1590.00	7.51	34.79	0.06	122.30
S019-150	150	47.37	-90.85	34.26	147.15	7.87	9.34	0.04	2.55	0.06	17.25	95.05	1580.00	6.41	34.43	0.06	121.05
S019-2m off bottom	2	47.37	-90.85	3.49	175.56	7.86	9.18	0.04	2.75	0.06	17.17	94.48	1580.00	7.03	35.79	0.05	141.55
S019-10m off bottom	10	47.37	-90.85	13.46	166.72	7.88	9.30	0.04	2.56	0.06	17.25	94.99	1590.00	6.15	33.57	0.06	118.04
S114-5	5	46.91	-86.60	98.61	4.90	7.92	9.21	0.04	2.79	0.06	17.15	95.92	1490.00	2.97	32.79	0.07	90.21
S114-10	10	46.91	-86.60	98.58	9.90	7.93	9.22	0.04	2.79	0.06	17.15	95.89	1490.00	3.08	29.93	0.08	72.85
S114-20	20	46.91	-86.60	98.61	19.63	7.95	9.22	0.04	2.77	0.06	17.16	95.84	1480.00	5.64	28.64	0.05	113.30
S114-50	50	46.91	-86.60	50.62	49.07	7.95	9.22	0.04	2.78	0.06	17.15	95.87	1480.00	5.79	28.36	0.07	85.46

S114-100	100	46.91	-86.60	98.61	98.10	7.95	9.22	0.04	2.80	0.06	17.15	95.94	1470.00	6.08	29.71	0.06	98.98
S114-150	150	46.91	-86.60	98.61	147.11	7.95	9.24	0.04	2.87	0.06	17.12	95.63	1460.00	6.63	30.57	0.08	77.39
S114-200	200	46.91	-86.60	98.61	196.29	7.96	9.24	0.04	2.88	0.06	17.11	95.71	1450.00	6.34	29.86	0.07	85.89
S114-250	250	46.91	-86.60	98.61	245.17	7.97	9.24	0.04	2.91	0.06	17.10	95.51	1220.00	6.89	28.00	0.10	59.07
S114-300	300	46.91	-86.60	92.06	294.26	7.97	9.25	0.04	2.97	0.06	17.07	95.28	1390.00	7.29	31.43	0.09	71.04
S114-365	365	46.91	-86.60	24.20	357.99	7.98	9.25	0.04	2.97	0.06	17.07	95.25	1030.00	7.03	na	na	na

na: data not available.

Table S2. Accession numbers, Number of base pairs and reads for each sampling sites for Lake Michigan and Lake Superior.

Sample Name	Lake	Sampling Station	Depth (m)	MG-RAST ID	No. of bp	No. of Reads
M028-5	Michigan	M028	5	4503845.3	4,079,416	27,016
M028-10	Michigan	M028	10	4503840.3	3,408,070	22,570
M028-20	Michigan	M028	20	4503843.3	3,111,053	20,603
M028-50	Michigan	M028	50	4503846.3	3,448,236	22,836
M028-100	Michigan	M028	100	4503841.3	3,958,465	26,215
M028-10m off bottom	Michigan	M028	10m off bottom	4503842.3	2,850,427	18,877
M028-2m off bottom	Michigan	M028	2m off bottom	4503844.3	3,901,085	25,835
M041-5	Michigan	M041	5	4503854.3	1,921,324	12,724
M041-10	Michigan	M041	10	4503847.3	1,583,235	10,485
M041-20	Michigan	M041	20	4503851.3	1,839,633	12,183
M041-50	Michigan	M041	50	4503855.3	1,140,956	7,556
M041-100	Michigan	M041	100	4503848.3	1,408,075	9,325
M041-10m off bottom	Michigan	M041	10m off bottom	4503849.3	1,589,577	10,527
M041-2m off bottom	Michigan	M041	2m off bottom	4503853.3	1,552,129	10,279
M041-150	Michigan	M041	150	4503850.3	1,480,857	9,807
M041-200	Michigan	M041	200	4503852.3	1,535,519	10,169
S001-5	Superior	S001	5	4503860.3	2,840,310	18,810
S001-10	Superior	S001	10	4503856.3	981,349	6,499
S001-20	Superior	S001	20	4503858.3	622,271	4,121
S001-50	Superior	S001	50	4503861.3	3,657,673	24,223
S001-10m off bottom	Superior	S001	10m off bottom	4503857.3	2,861,752	18,952
S001-2m off bottom	Superior	S001	2m off bottom	4503859.3	4,555,670	30,170
S008-5	Superior	S008	5	4503869.3	2,878,664	19,064
S008-10	Superior	S008	10	4503862.3	2,376,891	15,741
S008-20	Superior	S008	20	4503866.3	3,766,393	24,943
S008-50	Superior	S008	50	4503870.3	2,101,316	13,916
S008-100	Superior	S008	100	4503863.3	3,094,141	20,491
S008-10m off bottom	Superior	S008	10m off bottom	4503864.3	2,796,520	18,520
S008-2m off bottom	Superior	S008	2m off bottom	4503868.3	3,219,169	21,319
S008-150	Superior	S008	150	4503865.3	4,282,964	28,364
S008-200	Superior	S008	200	4503867.3	3,368,659	22,309
S019-5	Superior	S019	5	4503877.3	3,755,974	24,874
S019-10	Superior	S019	10	4503871.3	1,693,314	11,214
S019-20	Superior	S019	20	4503875.3	2,928,645	19,395
S019-50	Superior	S019	50	4503878.3	3,680,172	24,372
S019-100	Superior	S019	100	4503872.3	3,071,793	20,343
S019-10m off bottom	Superior	S019	10m off bottom	4503873.3	3,200,898	21,198
S019-2m off bottom	Superior	S019	2m off bottom	4503876.3	3,320,490	21,990
S019-150	Superior	S019	150	4503874.3	4,569,713	30,263
S114-5	Superior	S114	5	4503887.3	3,175,832	21,032
S114-10	Superior	S114	10	4503879.3	4,470,053	29,603
S114-20	Superior	S114	20	4503882.3	4,065,524	26,924
S114-50	Superior	S114	50	4503888.3	2,806,335	18,585
S114-100	Superior	S114	100	4503880.3	3,345,556	22,156
S114-150	Superior	S114	150	4503881.3	4,048,763	26,813
S114-200	Superior	S114	200	4503883.3	3,603,011	23,861
S114-250	Superior	S114	250	4503884.3	3,029,815	20,065
S114-300	Superior	S114	300	4503885.3	3,563,600	23,600
S114-365	Superior	S114	365	4503886.3	3,541,252	23,452

Table S3. Significantly different phyla in Lake Michigan and Lake Superior. Phyla above 1% abundance in either group are shown.

Phylum	LM	LS
Thaumarchaeota**	0.2	0.6
Bacteroidetes***	44.7	32.9
Proteobacteria	9.3	8.2
Cyanobacteria***	4.1	14.0
Verrucomicrobia***	7.3	13.9
Firmicutes	0.5	0.4
Actinobacteria***	0.7	3.1
Planctomycetes	0.8	0.6
Unclassified Bacteria	32.1	26.0
Other phyla	0.4	0.3

Table S4. Significantly different genera in Lake Michigan and Lake Superior. Genera above 1% abundance in either group are shown.

Phylum	Genus	LM	LS
Actinobacteria	Mycobacterium***	0.0	2.4
Bacteroidetes	Tenacibaculum*	10.5	8.1
Bacteroidetes	Terrimonas*	9.5	4.4
Bacteroidetes	Flavobacterium	5.8	7.9
Bacteroidetes	Alistipes	5.2	5.9
Bacteroidetes	unclassified Sphingobacteriaceae***	4.6	0.5
Bacteroidetes	Candidatus Amoebophilus**	3.0	1.4
Bacteroidetes	Hymenobacter*	2.1	1.1
Bacteroidetes	Chitinophaga***	0.9	0.3
Bacteroidetes	Marinoscillum***	0.6	1.2
Cyanobacteria	Synechococcus***	3.2	13.3
Proteobacteria	unclassified Comamonadaceae	4.2	3.9
Proteobacteria	unclassified Alphaproteobacteria***	1.0	0.5
Proteobacteria	unclassified Betaproteobacteria*	0.4	1.1
Verrucomicrobia	Diplosphaera	2.9	2.9
Verrucomicrobia	Chthoniobacter***	2.5	6.1
Verrucomicrobia	Pedosphaera***	0.7	3.5
Verrucomicrobia	Verrucomicrobium***	0.3	1.2
unclassified bacteria	unclassified bacteria	23.9	20.9

Table S5. Significantly different microbial phylum in Lake Michigan and Superior sampling sites.

Phylum	M028	M041	S001	S008	S019	S114
Thaumarchaeota***	0.11	0.25	0.27	1.26	0.42	0.56
Bacteroidetes***	53.61	45.11	42.70	30.47	33.90	34.67
Proteobacteria	8.78	11.10	5.49	11.18	7.17	9.36
Cyanobacteria***	5.51	3.67	12.36	13.65	18.09	14.59
Verrucomicrobia***	5.82	9.61	14.28	13.91	14.68	15.59
Firmicutes	0.17	0.87	0.84	0.24	0.40	0.39
Actinobacteria***	0.44	0.98	3.34	3.95	2.62	3.13
Planctomycetes***	0.29	1.37	0.72	0.92	0.56	0.38
unclassified bacteria	25.24	26.94	19.94	24.32	22.11	21.26
Other phyla	0.04	0.09	0.05	0.10	0.05	0.06

Other phyla include bacterial phyla below 0.01% abundance and are combined together (Chrysiogenetes, Chlamydiae, Gemmatimonadetes, Chlorobi, Nitrospirae, Poribacteria, Acidobacteria, Spirochaetes, Tenericutes, Deinococcus-Thermus, Fusobacteria). The significant differences among the abundance values in phyla was computed using One Way ANOVA with Tukey's post hoc test for multiple comparisons ***p <0.0001.

Table S6. Significantly different microbial genera in Lake Michigan and Superior sampling sites.

Genus	M028	M041	S001	S008	S019	S114	*p*-values	*p*-values (FDR corrected)
Terrimonas	15.15	6.55	5.88	4.26	4.30	4.72	0.00	0.00
Tenacibaculum	12.95	10.31	7.82	7.70	10.77	8.16	0.00	0.00
Flavobacterium	7.99	5.13	16.62	1.97	7.64	9.52	0.00	0.00
Synechococcus	4.60	2.64	11.81	13.03	17.23	13.68	0.00	0.00
Alistipes	4.06	6.88	4.89	10.54	4.78	4.50	0.00	0.00
unclassified Comamonadaceae	3.70	5.26	1.39	5.69	2.72	5.30	0.39	0.83
unclassified Sphingobacteriaceae	3.45	6.33	0.63	0.51	0.31	0.63	0.00	0.00
Hymenobacter	2.95	1.82	1.49	0.83	1.02	1.24	0.00	0.00
Diplosphaera	2.59	3.63	2.51	3.22	3.18	3.23	0.22	0.69
Candidatus Amoebophilus	2.46	3.98	1.19	1.57	1.42	1.49	0.00	0.00
Chthoniobacter	2.10	3.24	7.26	5.10	6.46	6.98	0.00	0.00
unclassified Alphaproteobacteria	1.21	1.06	0.57	0.55	0.54	0.53	0.00	0.00
Acidovorax	0.95	0.16	0.54	0.28	0.05	0.08	0.39	0.83
Chitinophaga	0.95	0.94	0.44	0.19	0.33	0.42	0.00	0.00
Flectobacillus	0.78	0.88	0.67	0.32	0.53	0.69	0.00	0.02
Castellaniella	0.54	0.29	0.00	0.00	0.00	0.00	0.03	0.15
Marinoscillum	0.51	0.70	1.70	1.19	1.09	1.09	0.00	0.02
Sphingobacterium	0.51	0.01	0.00	0.00	0.00	0.01	0.27	0.74
Mycobacterium	0.02	0.03	2.68	3.24	2.07	2.21	0.00	0.00
Nitrosopumilus	0.11	0.25	0.27	1.26	0.42	0.56	0.00	0.00
Pedosphaera	0.37	0.98	2.75	5.16	3.41	3.22	0.00	0.00
Verrucomicrobium	0.34	0.24	1.65	0.19	1.47	1.93	0.00	0.00

Table S7. Spearman Rank correlation among environmental parameters and major phyla in lake samples.

**Parameters**	**Altitude**	**Pressure**	**Depth**	**Latitude**	**Longitude**	**pH**	**Salinity**	**Conductivity**	**Fluorescence**	**DO**	**Nitrogen**	**Beam transmission**	**Surface irradance**	**Temperature**	**Nitrate**	**Total phosphorus**	**N:P ratio**
Altitude	0.00	0.00	0.27	0.79	0.96	0.95	0.88	0.87	0.61	0.26	0.66	0.28	0.54	0.65	0.54	0.02	0.70
Pressure	-0.50	0.00	0.00	0.52	0.98	0.62	0.65	0.67	0.76	0.00	0.26	0.09	0.80	0.25	0.01	0.29	0.73
Depth	0.16	**0.63**	0.00	0.38	0.71	0.67	0.30	0.31	0.15	0.72	0.84	0.23	0.59	0.85	0.00	0.88	0.07
Latitude	0.04	0.09	0.13	0.00	0.09	0.00	0.00	0.00	0.00	0.30	0.00	0.00	0.00	0.00	0.26	0.66	0.47
Longitude	0.01	0.00	0.05	-0.24	0.00	0.43	0.20	0.20	0.27	0.31	0.05	0.55	0.00	0.05	0.13	0.20	0.01
ph	-0.01	0.07	0.06	**-0.94**	0.12	0.00	0.00	0.00	0.01	0.13	0.00	0.00	0.10	0.00	0.11	0.98	0.29
Salinity	-0.02	-0.07	-0.15	**-0.97**	0.19	**0.92**	0.00	0.00	0.03	0.03	0.00	0.00	0.00	0.00	0.53	0.79	0.24
Conductivity	-0.02	-0.06	-0.15	**-0.97**	0.19	**0.92**	**1.00**	0.00	0.03	0.03	0.00	0.00	0.00	0.00	0.52	0.78	0.23
Fluorescence	-0.07	-0.04	0.21	-0.40	0.16	0.35	0.31	0.31	0.00	0.00	0.62	0.00	0.01	0.65	0.22	0.73	0.10
DO	0.16	-0.41	0.05	0.15	-0.15	-0.22	-0.31	-0.31	**0.53**	0.00	0.00	0.00	0.43	0.00	0.97	0.87	0.11
Nitrogen	0.07	-0.16	0.03	**0.87**	-0.28	**-0.88**	**-0.91**	**-0.92**	-0.07	**0.60**	0.00	0.03	0.03	0.00	0.20	0.77	0.24
Beam transmission	0.16	0.24	0.18	**0.70**	0.09	**-0.56**	**-0.57**	**-0.57**	**-0.65**	-0.47	0.31	0.00	0.07	0.04	0.55	0.53	0.90
Surface irradance	0.09	0.04	0.08	0.40	-0.47	-0.23	-0.45	-0.45	-0.38	0.12	0.31	0.26	0.00	0.03	0.41	0.26	0.04
Temperature	-0.07	0.17	-0.03	**-0.86**	0.28	**0.87**	**0.91**	**0.91**	0.07	**-0.60**	**-1.00**	-0.30	-0.30	0.00	-0.19	0.20	0.76	0.24
Nitrate	0.09	-0.36	**-0.52**	0.16	-0.22	-0.23	-0.09	-0.09	-0.18	0.01	0.19	0.09	0.12	-0.19	0.00	0.06	0.00
Total phosphorus	0.33	-0.15	-0.02	0.06	-0.19	0.00	-0.04	-0.04	-0.05	0.02	0.04	0.09	0.16	-0.04	0.27	0.00	0.94
N:P ratio	-0.06	-0.05	-0.26	-0.11	-0.37	0.16	0.17	0.17	-0.24	-0.23	-0.17	-0.02	0.29	0.17	**0.49**	0.01	0.00
Thaumarchaeota	0.01	0.25	0.12	**0.63**	0.01	**-0.53**	**-0.54**	**-0.53**	**-0.61**	-0.42	0.26	**0.82**	**0.50**	-0.25	0.08	-0.05	0.05
Proteobacteria	-0.16	0.27	-0.01	-0.09	0.07	0.13	0.15	0.15	-0.32	-0.48	-0.29	0.25	0.06	0.30	0.06	-0.12	0.25
Firmicutes	-0.05	-0.06	-0.23	-0.07	0.14	-0.01	0.11	0.11	0.02	-0.17	-0.06	0.02	-0.44	0.06	0.06	-0.02	-0.01
Bacteroidetes	-0.09	-0.20	-0.15	**-0.83**	0.29	**0.69**	**0.76**	**0.75**	0.48	0.10	**-0.60**	**-0.75**	-0.45	**0.59**	-0.16	-0.13	0.00
Actinobacteria	0.00	0.02	-0.06	**0.79**	0.03	**-0.78**	**-0.78**	**-0.78**	-0.44	-0.05	**0.62**	**0.68**	0.32	**-0.62**	0.20	-0.05	0.01
Cyanobacteria	0.27	-0.19	0.13	**0.75**	-0.40	**-0.68**	**-0.78**	**-0.78**	-0.07	**0.59**	**0.86**	0.30	**0.51**	**-0.87**	0.18	0.14	-0.05
Verrucomicrobia	0.04	0.25	0.29	**0.85**	-0.16	**-0.77**	**-0.84**	**-0.84**	-0.32	0.10	**0.72**	**0.61**	0.21	**-0.72**	-0.06	0.05	-0.32
Planctomycetes	-0.07	0.16	-0.14	-0.07	0.12	0.04	0.23	0.24	-0.19	**-0.62**	-0.32	0.28	-0.43	0.32	0.07	-0.06	-0.10
Unclassified bacteria	-0.05	0.11	-0.06	-0.30	0.01	0.35	0.36	0.36	-0.02	-0.36	-0.44	-0.07	-0.10	0.44	-0.03	0.13	0.07
Other phyla	-0.10	0.16	-0.17	0.05	0.09	-0.06	0.03	0.03	-0.42	**-0.62**	-0.23	0.37	-0.06	0.24	0.00	-0.07	0.02

Spearman rank correlation (r) values above 0.45 are highlighted in bold.

p values < 0.05 are highlighted.
