# Supplementary material for: Aqueous Geochemical Controls on the Sestonic Microbial Community in Lakes Michigan and Superior
Source: Microorganisms. 2023 Feb 17;11(2):504. doi: 10.3390/microorganisms11020504 (PMC9963676; doi:10.3390/microorganisms11020504)
Supplement: Supplementary file 1 [file microorganisms-11-00504-s001.zip › microorganisms-2128228 -S3.pdf]

**SUPPLEMENTARY DATA**

# Aqueous Geochemical Controls on the Sestonic Microbial Community in Lakes Michigan and Superior

Asha Rani[1, *], Ravi Ranjan[2], Solidea M.C. Bonina[1], Mahsa Izadmehr[1, #], John P. Giesy[3,4, 5], An Li[6], Neil C. Sturchio[7], Karl J. Rockne[1, **]

[1] Department of Civil, Materials, and Environmental Engineering, University of Illinois at Chicago, Chicago, Illinois 60607, USA

[2] Genomics Resource Laboratory, Institute for Applied Life Sciences (IALS), University of Massachusetts, Amherst, 01003, USA

[3] Department of Veterinary Biomedical Sciences and Toxicology Centre, University of Saskatchewan, Saskatoon, Saskatchewan S7N 5C5, Canada

[4] Department of Environmental Science, Baylor University, One Bear Place 97266, Waco, TX 76706, USA

[5] Department of Zoology and Center for Integrative Toxicology, Michigan State University, East Lansing, MI, USA

[6] School of Public Health, University of Illinois at Chicago, Chicago, Illinois 60612, USA

[7] Department of Earth Sciences, University of Delaware, Newark, Delaware, 19716, USA

[*] Current address: Department of Food Science, University of Massachusetts, Amherst, 01003, USA

[#] Current address: California Regional Water Quality Control Board, San Diego Region, San Diego, California, 92108

** Corresponding author: phone: 1-312-413-0391; fax: 1-312-996-2423; e-mail: krockne@uic.edu

## MATERIALS AND METHODS

### *Sampling*

At each location, a rosette sampling system was deployed from surface to the bottom with Van Dorn samplers. At each selected sampling depth, 1 L of water sample from the rosette sampler was collected and filtered on sterile filters (Whatman 0.22 μm filter, Millipore Sigma) for microbial analysis. All samples were aseptically transferred to a freezer (-40 °C) until DNA extraction in the laboratory. Samples of water were frozen at -20 °C prior to subsequent analysis in the laboratory. A Sea-Bird multi-sensor profiling instrument was attached to the rosette to collect conductivity, water pressure, temperature, depth, DO, pH, light penetration, water transparency, and chlorophyll a data continuously from the surface to bottom. Samples of sediment were obtained at all sampling locations as part of a large study on pollutants in the Great Lakes [1-9]. Filtered samples were analyzed for nitrate-N and total phosphorus (TP) by automated analysis (AQ300 autoanalyzer, SEAL Analytical, Mequon, WI) using EPA-114-A Rev.11 (for $NO_3$+$NO_2$) and EPA-155-D Rev.0 (for soluble reactive phosphorus, SRP) as described previously [10]. True value standards and replicates were performed at least once out of every ten analytical samples for QA/QC purposes. Bias (*B*) was determined from the true value standards and precision from the standard deviation of replicates ($\sigma$). From these the total analytical uncertainty at the 95% confidence interval (CI) was determined using $U_{95}$ = SQRT($B^2$ + $2\sigma^2$) according to Standard Methods [11]. Given the very small concentrations of phosphorus in these samples, only the lowest true value standard was used for determining the bias to be most representative of the concentration range in these samples.

### *Extraction, PCR amplification and Sequencing of DNA*

DNA was quantified by a Qubit 2.0 Fluorometer (Life Technologies, Carlsbad, CA, USA). Control samples yielded DNA that was below detection limits (<0.5 ng/mL). Amplicon PCR was performed on the V4 region of the 16S rRNA gene using the primer pair 515F–806R (Golay error-correcting barcodes on the reverse primer [12]). This primer pair is best known for sampling both bacteria and non-eukaryotic archaea [13]. Amplicons were barcoded and pooled in equal concentrations for sequencing. The amplicon pool was purified with the MoBio UltraClean PCR Clean-up kit (Qiagen Inc, USA) and sequenced on the Illumina HiSeq sequencing platform using a paired end 151 bp read chemistry. All samples were sequenced at Argonne National Laboratory (Lemont, Illinois, USA). The detailed methods are provided in the EMP protocols website (http://www.earthmicrobiome.org/) and elsewhere [14,15]. Sequence data were demultiplexed and

quality filtered using the QIIME 1.9.1 script split_libraries_fastq.py with default parameters to generate FASTA sequence files [16].

### *Bioinformatics and statistical analysis*

Data were confirmed to follow a normal probability distribution using Shapiro-Wilkes and variance homogeneity was confirmed by Levene's test using statistical software (SYSTAT v13, InPixon Inc., Palo Alto, CA USA). Multiple group comparisons were assessed by analysis of variance (ANOVA) followed by Tukey's *post hoc* tests. Statistical analysis for one-way ANOVA, correlation analysis, and multiple test corrections for group comparisons was carried out using GraphPad prism (GraphPad Prism v.7.0, GraphPad Software, San Diego, CA, USA). Data were considered as statistically significant with $p<0.05$ unless otherwise indicated. Differences in lake microbial diversity, sampling locations, and depths were analyzed through principal coordinate analysis (PCoA) to find the axis that best explains the variance in the data set. The PCoA plot was generated using the normalized data matrix for phylum and genus level OTU in each sample. Cluster analysis was performed using Pearson correlation distance with Ward method between the lakes at phylum and genus level using METAGENassist [17]. Linear models and ANOVA were used to test for differences in alpha diversity between the lakes. Alpha diversity values were correlated with environmental variables using Spearman's rank correlation. Canonical Correspondence Analysis (CCA) was implemented to explore relationships between environmental variables significantly correlated to microbial community beta diversity. Permutation tests were implemented to test significance of environmental variables within CCA in explaining beta diversity patterns using 999 permutations. Spearman's Rank correlations were used to identify OTUs significantly correlated to environmental variables (DO, pH, temperature, salinity, etc.). Similarity percentage (SIMPER) analysis was used to identify the taxa that were responsible for the differences observed across lakes and sampling locations. Analysis of Similarity (ANOSIM) was used to test for significant differences in compositions of microbial communities between lakes and sampling locations. All analyses were conducted using the Bray-Curtis distance matrix using the statistical software package PAST 3.18b [18]. A table of OTUs and metadata files were further analyzed with the Calypso web-server [19] for feature selection, linear discriminant analysis (LDA) effect size (LEfSe) analysis (log odds ratio score plots), diversity indices, and microbial network analysis. L*DA based LEfSe approach* was used to understand the differences in two or more groups. Taxonomic read count data were used as the input and output files were saved. LEfSe identifies taxa that best explain the differences in two or more groups. We performed the analysis with lake type (LM versus LS) and across sampling

locations as different groups. LEfSe determined a biomarker signature composed of bacterial phyla/genera preferentially associated with a given lake or sampling location. Biomarker taxa from one sample could also be present in another sample but were either detected in small abundances or were absent.

***Microbial Network Analysis***

Network analysis was carried out for identifying clusters of co-occurring bacterial taxa and environmental variables in both lakes. Pearson's correlation network analysis was performed in Calypso version 8.4 (http://bioinfo.qimr.edu.au/calypso/). Taxa and environmental variables are represented as nodes, taxa abundance as node size, and edges represent positive and negative associations. Nodes are color coded by the taxa and taxa abundances are associated with environmental variables using Pearson's correlation index. Nodes are colored based on the strength of the association with each environmental variable. The resulting pairwise correlations were converted into dissimilarities and were used to ordinate nodes in a two-dimensional plot by PCoA. The correlating nodes are in close proximity and anti-correlating nodes are distant in the network. P-values for Bray-Curtis dissimilarities were computed by 1000-fold permutation and corrected by False Discovery Rate (FDR). Significant associations (FDR $< 0.05$) are presented as edges. Bacterial genera (light blue) and environmental variables (red) are represented as nodes.

***Molecular phylogenetic analysis for Thaumarchaeota***

The evolutionary history was inferred using the neighbor-joining method and the evolutionary distances were computed using the maximum composite likelihood method [20]. All positions containing gaps were eliminated and high-quality alignments were used for reconstructions of phylogenetic trees. The bootstrap consensus tree inferred from 100 replicates was used to represent the evolutionary history of the taxa analyzed [21]. The bootstrap values given at nodes are percentages of 100 replicates. Candidatus *Nitrosopumilus sp*. NM25 (AB546961.1) was used as the reference Archaea (as the closest database match). *Sulfolobus solfataricus* (X03235.1) and *Escherichia coli* K-12 (S000529088) were used as the outgroup archaea and bacteria type strains, respectively. All nucleotide sequences were aligned using ClustalW and a phylogenetic tree was constructed using MEGA version 4.0 [22].

**RESULTS**

***Microbial community structure***

Microbial (Bacterial/Archaeal) 16S rRNA gene-based amplicon sequencing was applied to investigate the structures of microbial communities in both LM and LS. A total of 954,189 raw sequences were obtained from all samples from both lakes. After quality filtration steps, high quality sequences were included in the analysis, sample description and sequence details are provided in Table S2. Like the PCA analysis at phylum and genus level, sampling locations were also readily differentiated between lakes. The first two principal components explained 11.8% (7.7% + 4.1%) of the total variance at the genus level based on sampling location profile using a 95% confidence interval threshold. Species composition in LS sampling locations S001 and S008 showed more overlap compared to those in S019 and S114, which were distant from those in other locations (Figs. 4 & S3).

***Microbial taxa and diversity indices between Lakes***

Beyond major phyla, Firmicutes, Planctomycetes, and "other phyla" (which included 11 phyla that contributed less than 1% to the total abundance) comprised much of the microbiome in both lakes (Fig. 5A, Table S3). The top 19 genera/families comprising >1% abundance demonstrate that the genera *Synechococcus* (Cyanobacteria) was most abundant (13.3%) in LS, and *Tenacibaculum* (Bacteroidetes) in LM (10.5%). Genus *Terrimonas*, unclassified *Sphingobacteriaceae*, and unclassified *Comamonadaceae* were greater in abundance in LM, compared to *Flavobacterium* and *Chthoniobacter* in LS (Fig. 5B). Among the top 19 genera/families, 11 genera and 3 unclassified class/families were significantly different among the groups. Similar to trends observed at the phylum level, significant differences were observed at the genus level ($p$<0.0001) in α-diversity, Shannon diversity index, Simpson diversity index, and species evenness at various depths (Fig. S5). Shannon diversity index and species evenness values were greatest for LS compared to LM (Fig. S5). Our data show slightly greater microbial diversity in near bottom-water depth compared to surface waters. While these differences in alpha diversity may be driven by differences in temperature, pH, DO, salinity, and light penetration as shown by beam transmission, it is likely that light penetration plays the most important role as all locations with greater diversity (except S008) have the lowest beam transmittance in the profundal depths (Fig. 2).

***Microbial community structure in different sampling locations***

A detailed description of the 22 most abundant bacterial genera is given in Fig. 6B. Each sampling location revealed a characteristic site-specific microbial profile that was readily distinguishable from other locations. For example, the genus *Terrimonas* (15%) and *Tenacibaculum* (13%), and *Hymenobacter* (3%) were comparably greater at site M028 compared to all other locations. The genus *Synechococcus* was relatively low in LM (2.6-4.6%) compared to all LS locations, where it comprised 12-17% of all genera. Unclassified *Sphingomonadaceae* was greater (4-6%) in all LM locations than in all LS locations (0.3-0.6%). The Archaeal genera *Nitrosopumilus* (1.3%) was greatest in sampling location S008 in LS (Fig. 6B, Table S6). The eight bacterial genera *Chthoniobacter*, *Flavobacterium*, *Hymenobacter*, *Nitrosopumilus*, *Opitutus*, *Pseudanabaena*, *Tenacibaculum*, and *Terrimonas* were predominant across sampling locations and depths in all sampling locations (Fig. S6). A Bray-Curtis matrix based on genus abundance profiles was used to generate a relative abundance heatmap of the 15 most abundant bacterial taxa across all depths (Fig. S14).

***Microbial community structure in different depths***

In M028, a great abundance of Proteobacteria and lesser abundance of Bacteroidetes were detected in samples from 2 m and 10 m off the bottom compared to samples within 10 m of the surface (Fig. S13). A great abundance of Bacteroidetes was detected at depths of 20, 50 and 100 m at M028 compared to both near bottom and surface water samples. The Archaeal phylum Thaumarchaeota was detected at greatest abundance (1.5-1.6%) in near bottom depths (200, 2 and 10 m off bottom) compared to surface water (10-20 m) samples and was greatest among the sampling locations. A site-specific profile was observed at the deepest depth at each site (up to 365 m), where typically the least concentrations of Proteobacteria and Cyanobacteria were observed (Figs. S13, S7G-H).

***Biomarker signature analysis (LEfSe)***

LEfSe analysis was used for statistical analysis of microbiome data to understand compositions of microbial classes at various sample locations through an analysis of the most discriminating bacterial groups within each sampling location and between lakes [23]. Phylum Bacteroidetes and Planctomycetes were enriched at sampling locations M028 and M041, respectively in LM. Phylum Firmicutes and Cyanobacteria were predominant in the sampling locations S001 and S019, respectively in LS. *Thaumarchaeota* and Actinobacteria were statistically significantly discriminating phyla in sampling location S008 in LS (Fig. S9A & B). Genera *Terrimonas*,

*Tenacibaculum*, *Hymenobacter*, and unclassified Alphaproteobacteria were biomarker genera for site M028 in LM and unclassified *Sphingobacteriaceae*, *Candidatus Amoebophilus*, *Chitinophaga*, *Flactobacillus*, and *Planctomycetes* were predominant at LM site M041. In LS, *Flavobacterium*, and *Marinoscillum* were discriminant genera for sampling location S001. *Alistipes*, *Nitrosopumilus*, and unclassified Deltaproteobacteria were enriched taxa for LS sampling site S008. *Synechocococcus*, *Prolixibacter*, and *Thioalkalivibrio* were biomarker genera for S019 site in LS. Finally, *Chthoniobacter*, *Pseudanabena*, and unclassified Betaproteobacteria were characteristics genera for site S114 in LS (Fig. S9B). In LM, biomarker genera were *Terrimonas*, *Tenacibaculum*, *Hymenobacter*, *Chitinophaga*, *Rathayibacter*, *Acidovorax*, *Flectobacillus*, *Chryseobacterium*, and unclassified members of *Sphingobacteriaceae*, alpha and gamma-proteobacteria among others. In LS, genera *Synechococcus*, *Chthoniobacter*, *Marinoscillum*, *Terrimonas*, and *Nitrosopumilus* were significantly abundant at sampling location comparisons between lakes.

### *Distribution of Thaumarchaeota*

A phylogenetic analysis identified *Candidatus Nitrosopumilus* as the most closely-related (97% similarity) *Thaumarchaeota* in LM and LS locations (Fig. S8). In LM sampling location M028, least abundance of *Thaumarchaeota* (0.1-0.2%) was observed among depths compared to location M041 (0.2-0.6%) (Fig. 7A-B). The composition at LS sampling location S001 had a similar proportion (0.2-0.7%) of *Thaumarchaeota* as at location M041. Both locations had the greatest abundance of this phylum at depths of 10-20 m.

### *Environmental variation and community structure*

ANOSIM between lakes and among sampling locations identified key differences in structures of microbial communities (Fig. S11A, Fig S11B; ANOSIM R = 0.72, $p$<0.0001). Physicochemical variables found to be significantly correlated with beta diversity in CCA were subsequently tested as constraining variables on beta diversity (Fig. 8A-B). On the horizontal axis (CCA1), the most influential physicochemical variables were temperature, conductivity, salinity (biplot score = −0.9), latitude, $N_2$ saturation (biplot score = 0.9) and pH (biplot score = −0.8), followed by beam transmission (biplot score = 0.6), surface irradiance (biplot score = 0.5), and longitude (biplot score = 0.3) (Fig. 8B). On the vertical axis (CCA2), the most influential physicochemical variables were DO (biplot score = -0.8), beam transmission (biplot score = 0.7), fluorescence (biplot score = -0.6), and pressure (biplot score = 0.4) (Fig. 8B). A strong influence of latitude (biplot score = ±0.2) was not observed across both axes. DO, temperature, salinity and pH were significant

constraints on microbial community beta diversity ($p<0.001$), as compared to pressure and latitude; even when controlling for mutual dependence between parameters. For example, latitude has some correlation with temperature, but changes in temperature were greater with depth than by latitude. Results of studies have shown that latitude offers a strong biogeographical gradient and can significantly relate to changes in freshwater lake microbial community structure [24]. However, $N_2$ saturation, temperature, pH, DO, salinity, and other factors vary with latitude and interact and influence ecological communities, thus these parameters are more likely to affect microbial diversity rather than latitude alone [25,26].

***Microbial relationships to environmental variables and Network Analysis***

A Pearson-correlation-based network analysis revealed a positive correlation between an increased abundance of *Terrimonas*, *Hymenobacter*, *Chitinophaga*, unclassified *Sphingobacteriaceae*, and *Candidatus Amoebophilus* with pH, salinity, temperature, and conductivity (Fig. S12). Greater abundances of *Flavobacterium*, *Tenacibaculum*, and unclassified Flavobacteria were positively correlated with DO, altitude, and fluorescence, while *Nitrosopumilus*, *Pseudomonas*, *Synechococcus*, *Chthoniobacte*r, *Marinoscillum*, *Chthoniobacter* abundance was positively correlated with $N_2$ saturation, DO, latitude, beam transmission, and surface irradiance (Fig. S12).

## References

1. Corcoran, M.; Sherif, M.I.; Smalley, C.; Li, A.; Rockne, K.J.; Giesy, J.P.; Sturchio, N.C. Accumulation rates, focusing factors, and chronologies from depth profiles of 210Pb and 137Cs in sediments of the Laurentian Great Lakes. *J. Great Lakes Res.* **2018**, *44*, 693-704, doi:https://doi.org/10.1016/j.jglr.2018.05.013.
2. Bonina, S.M.C.; Codling, G.; Corcoran, M.B.; Guo, J.; Giesy, J.P.; Li, A.; Sturchio, N.C.; Rockne, K.J. Temporal and spatial differences in deposition of organic matter and black carbon in Lake Michigan sediments over the period 1850–2010. *J. Great Lakes Res.* **2018**, *44*, 705-715, doi:https://doi.org/10.1016/j.jglr.2018.03.001.
3. Codling, G.; Hosseini, S.; Corcoran, M.B.; Bonina, S.; Lin, T.; Li, A.; Sturchio, N.C.; Rockne, K.J.; Ji, K.; Peng, H.; et al. Current and historical concentrations of poly and perfluorinated compounds in sediments of the northern Great Lakes – Superior, Huron, and Michigan. *Environ. Pollut.* **2018**, *236*, 373-381, doi:https://doi.org/10.1016/j.envpol.2018.01.065.
4. Li, A.; Guo, J.; Li, Z.; Lin, T.; Zhou, S.; He, H.; Ranansinghe, P.; Sturchio, N.C.; Rockne, K.J.; Giesy, J.P. Legacy polychlorinated organic pollutants in the sediment of the Great Lakes. *J. Great Lakes Res.* **2018**, *44*, 682-692, doi:https://doi.org/10.1016/j.jglr.2018.02.002.
5. Guo, J.; Li, Z.; Ranasinghe, P.; Rockne, K.J.; Sturchio, N.C.; Giesy, J.P.; Li, A. Halogenated flame retardants in sediments from the Upper Laurentian Great Lakes: Implications to long-range transport and evidence of long-term transformation. *J. Hazard. Mater.* **2020**, *384*, 121346, doi:https://doi.org/10.1016/j.jhazmat.2019.121346.

6. Cao, D.; Guo, J.; Wang, Y.; Li, Z.; Liang, K.; Corcoran, M.B.; Hosseini, S.; Bonina, S.M.C.; Rockne, K.J.; Sturchio, N.C.; et al. Organophosphate Esters in Sediment of the Great Lakes. *Environ. Sci. Technol.* **2017**, *51*, 1441-1449, doi:10.1021/acs.est.6b05484.
7. Guo, J.; Li, Z.; Ranasinghe, P.; Bonina, S.; Hosseini, S.; Corcoran, M.B.; Smalley, C.; Rockne, K.J.; Sturchio, N.C.; Giesy, J.P.; et al. Spatial and Temporal Trends of Polyhalogenated Carbazoles in Sediments of Upper Great Lakes: Insights into Their Origin. *Environ. Sci. Technol.* **2017**, *51*, 89-97, doi:10.1021/acs.est.6b06128.
8. Peng, H.; Chen, C.; Cantin, J.; Saunders, D.M.V.; Sun, J.; Tang, S.; Codling, G.; Hecker, M.; Wiseman, S.; Jones, P.D.; et al. Untargeted Screening and Distribution of Organo-Bromine Compounds in Sediments of Lake Michigan. *Environ. Sci. Technol.* **2016**, *50*, 321-330, doi:10.1021/acs.est.5b04709.
9. Guo, J.; Li, Z.; Ranasinghe, P.; Bonina, S.; Hosseini, S.; Corcoran, M.B.; Smalley, C.; Kaliappan, R.; Wu, Y.; Chen, D.; et al. Occurrence of Atrazine and Related Compounds in Sediments of Upper Great Lakes. *Environ. Sci. Technol.* **2016**, *50*, 7335-7343, doi:10.1021/acs.est.6b00706.
10. Izadmehr, M.; Rockne, K. Pocket Wetlands" for nutrient removal in tile-drained agriculture. In Proceedings of the World Environmental and Water Resources Congress 2018: Watershed Management, Irrigation and Drainage, and Water Resources Planning and Management., 2018; pp. 404-414.
11. APHA-AWWA-WEF. *Standard methods for the examination of water and waste water*; American Public Health Association, American Water Works Association, Water Environment Federation, Washington DC: 2018.
12. Caporaso, J.G.; Lauber, C.L.; Walters, W.A.; Berg-Lyons, D.; Huntley, J.; Fierer, N.; Owens, S.M.; Betley, J.; Fraser, L.; Bauer, M.; et al. Ultra-high-throughput microbial community analysis on the Illumina HiSeq and MiSeq platforms. *The Isme Journal* **2012**, *6*, 1621, doi:10.1038/ismej.2012.8

https://www.nature.com/articles/ismej20128#supplementary-information.
13. Eloe-Fadrosh, E.A.; Ivanova, N.N.; Woyke, T.; Kyrpides, N.C. Metagenomics uncovers gaps in amplicon-based detection of microbial diversity. *Nature Microbiology* **2016**, *1*, 15032, doi:10.1038/nmicrobiol.2015.32

https://www.nature.com/articles/nmicrobiol201532#supplementary-information.
14. Thompson, L.R.; Sanders, J.G.; McDonald, D.; Amir, A.; Ladau, J.; Locey, K.J.; Prill, R.J.; Tripathi, A.; Gibbons, S.M.; Ackermann, G.; et al. A communal catalogue reveals Earth's multiscale microbial diversity. *Nature* **2017**, *551*, 457-463, doi:10.1038/nature24621.
15. Rani, A.; Rockne, K.J.; Drummond, J.; Al-Hinai, M.; Ranjan, R. Geochemical influences and mercury methylation of a dental wastewater microbiome. *Scientific Reports* **2015**, *5*, 12872, doi:10.1038/srep12872.
16. Caporaso, J.G.; Kuczynski, J.; Stombaugh, J.; Bittinger, K.; Bushman, F.D.; Costello, E.K.; Fierer, N.; Peña, A.G.; Goodrich, J.K.; Gordon, J.I.; et al. QIIME allows analysis of high-throughput community sequencing data. *Nat. Methods* **2010**, *7*, 335, doi:10.1038/nmeth.f.303

https://www.nature.com/articles/nmeth.f.303#supplementary-information.
17. Arndt, D.; Xia, J.; Liu, Y.; Zhou, Y.; Guo, A.C.; Cruz, J.A.; Sinelnikov, I.; Budwill, K.; Nesbø, C.L.; Wishart, D.S. METAGENassist: a comprehensive web server for comparative metagenomics. *Nucleic Acids Res.* **2012**, *40*, W88-W95, doi:10.1093/nar/gks497.
18. Hammer, O.; Haper, D.; Ryan, P. PAST: Paleontological statistics software package for education and data analysis. *Palaeontol Electron* **2001**, *4*, 9.

19. Zakrzewski, M.; Proietti, C.; Ellis, J.J.; Hasan, S.; Brion, M.-J.; Berger, B.; Krause, L. Calypso: a user-friendly web-server for mining and visualizing microbiome–environment interactions. *Bioinformatics* **2017**, *33*, 782-783, doi:10.1093/bioinformatics/btw725.
20. Tamura, K.; Nei, M. Estimation of the number of nucleotide substitutions in the control region of mitochondrial DNA in humans and chimpanzees. *Mol. Biol. Evol.* **1993**, *10*, 512-526.
21. Felsenstein, J. Phylogenies and the Comparative Method. *The American Naturalist* **1985**, *125*, 1-15, doi:10.1086/284325.
22. Tamura, K.; Dudley, J.; Nei, M.; Kumar, S. MEGA4: Molecular Evolutionary Genetics Analysis (MEGA) software version 4.0. *Mol. Biol. Evol.* **2007**, *24*, 1596-1599, doi:10.1093/molbev/msm092.
23. Segata, N.; Izard, J.; Waldron, L.; Gevers, D.; Miropolsky, L.; Garrett, W.S.; Huttenhower, C. Metagenomic biomarker discovery and explanation. *Genome Biology* **2011**, *12*, R60, doi:10.1186/gb-2011-12-6-r60.
24. Soininen, J.; Korhonen, J.J.; Luoto, M. Stochastic species distributions are driven by organism size. *Ecology* **2013**, *94*, 660-670.
25. Jones, S.E.; Newton, R.J.; McMahon, K.D. Potential for atmospheric deposition of bacteria to influence bacterioplankton communities. *FEMS Microbiol. Ecol.* **2008**, *64*, 388-394, doi:10.1111/j.1574-6941.2008.00476.x.
26. Sundqvist, M.K.; Sanders, N.J.; Wardle, D.A. Community and Ecosystem Responses to Elevational Gradients: Processes, Mechanisms, and Insights for Global Change. *Annual Review of Ecology, Evolution, and Systematics* **2013**, *44*, 261-280, doi:10.1146/annurev-ecolsys-110512-135750.
