# Supplementary material for: Aqueous Geochemical Controls on the Sestonic Microbial Community in Lakes Michigan and Superior
Source: Microorganisms. 2023 Feb 17;11(2):504. doi: 10.3390/microorganisms11020504 (PMC9963676; doi:10.3390/microorganisms11020504)
Supplement: Supplementary file 1 [file microorganisms-11-00504-s001.zip › microorganisms-2128228-S2.pptx]

## Slide 1
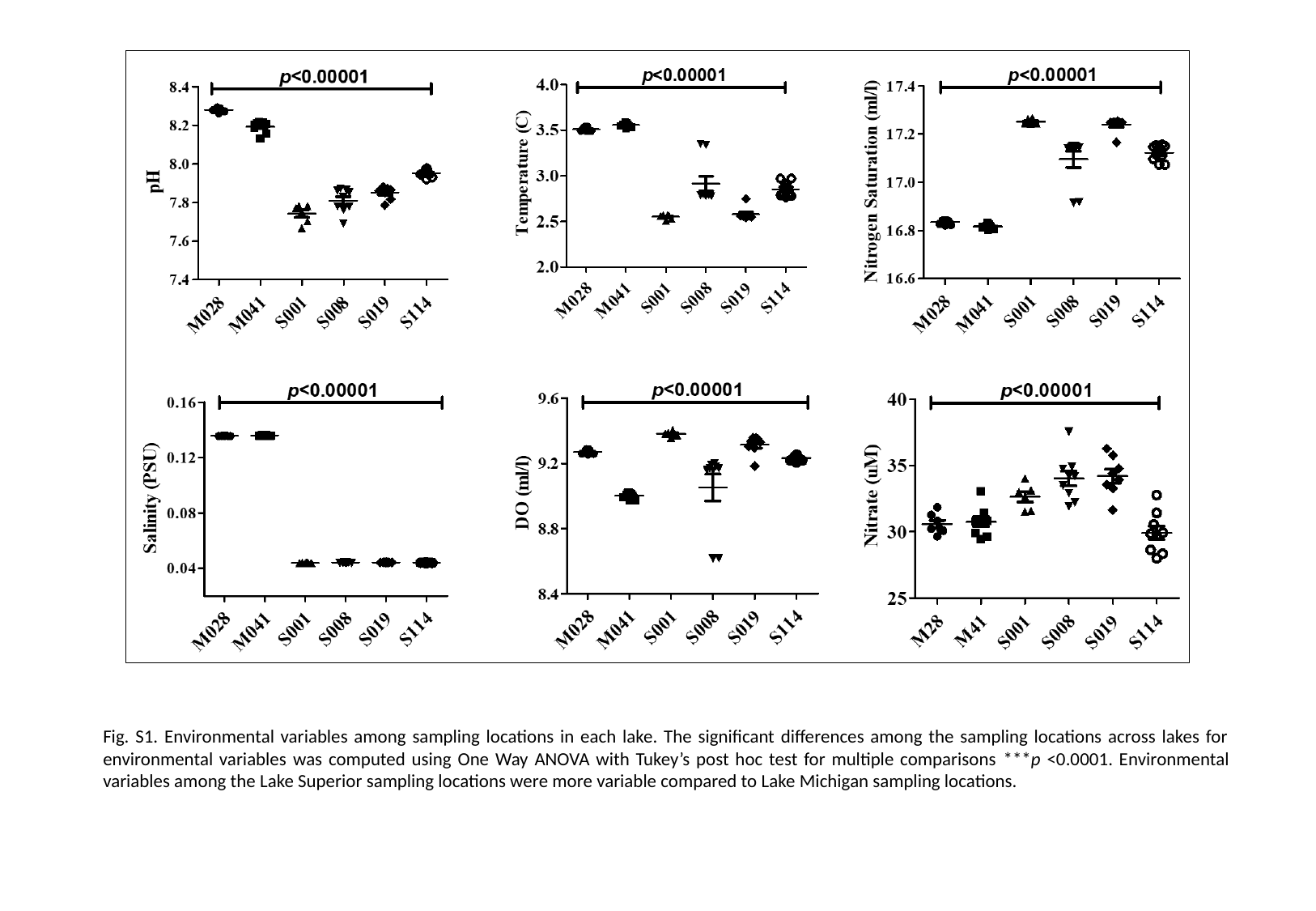

Fig. S1. Environmental variables among sampling locations in each lake. The significant differences among the sampling locations across lakes for environmental variables was computed using One Way ANOVA with Tukey’s post hoc test for multiple comparisons ***p <0.0001. Environmental variables among the Lake Superior sampling locations were more variable compared to Lake Michigan sampling locations.

## Slide 2
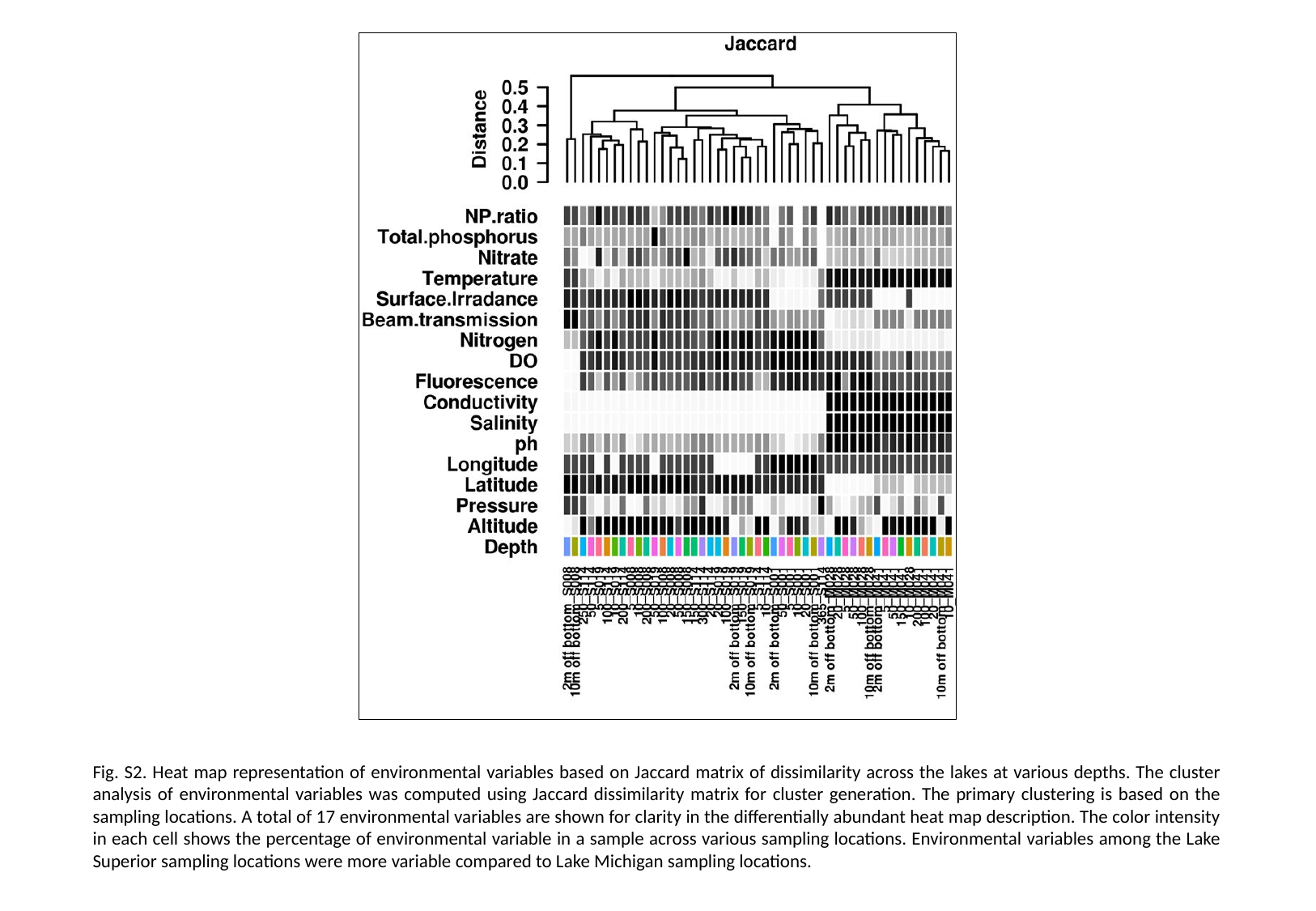

Fig. S2. Heat map representation of environmental variables based on Jaccard matrix of dissimilarity across the lakes at various depths. The cluster analysis of environmental variables was computed using Jaccard dissimilarity matrix for cluster generation. The primary clustering is based on the sampling locations. A total of 17 environmental variables are shown for clarity in the differentially abundant heat map description. The color intensity in each cell shows the percentage of environmental variable in a sample across various sampling locations. Environmental variables among the Lake Superior sampling locations were more variable compared to Lake Michigan sampling locations.

## Slide 3
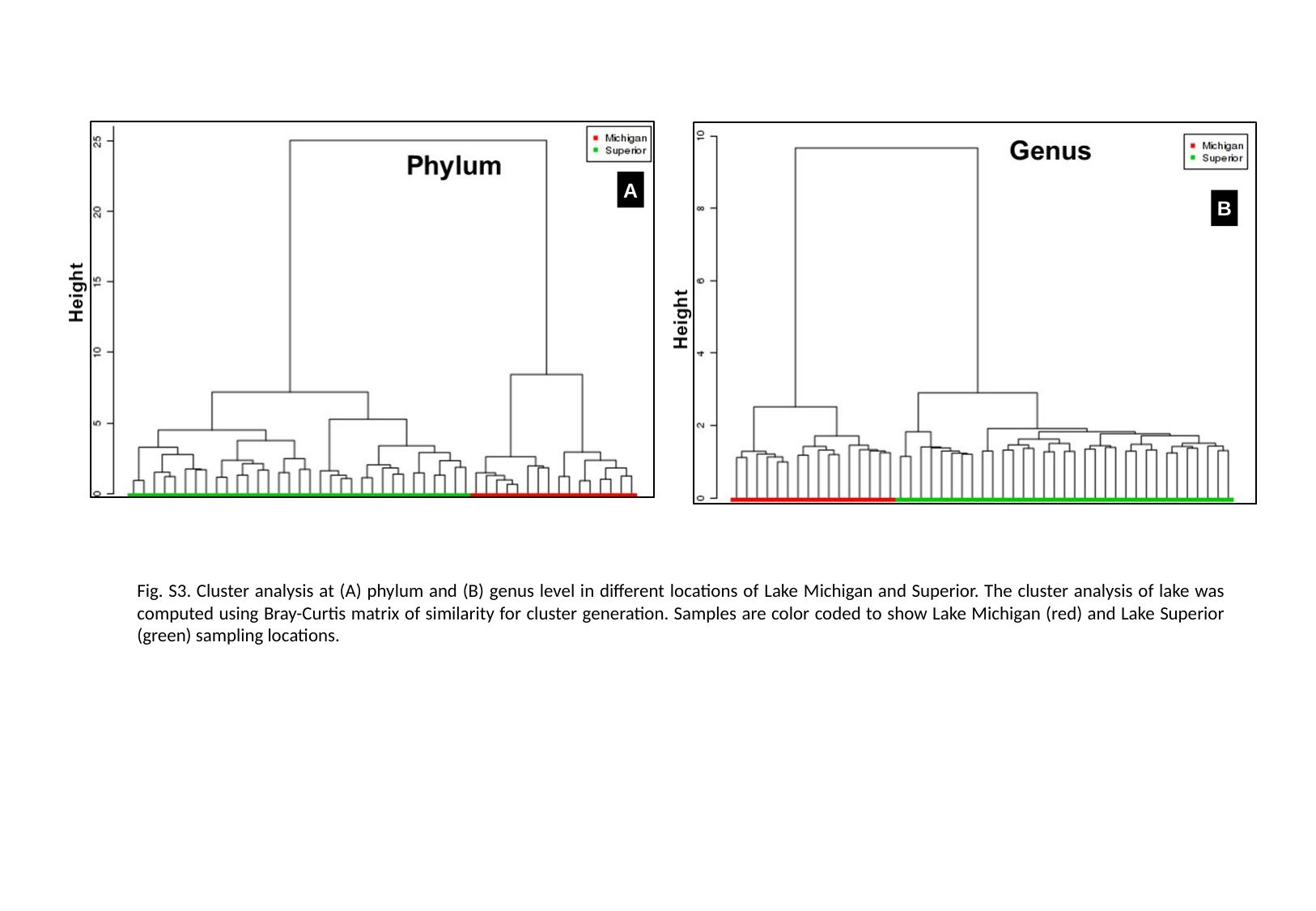

A
B
Fig. S3. Cluster analysis at (A) phylum and (B) genus level in different locations of Lake Michigan and Superior. The cluster analysis of lake was computed using Bray-Curtis matrix of similarity for cluster generation. Samples are color coded to show Lake Michigan (red) and Lake Superior (green) sampling locations.

## Slide 4
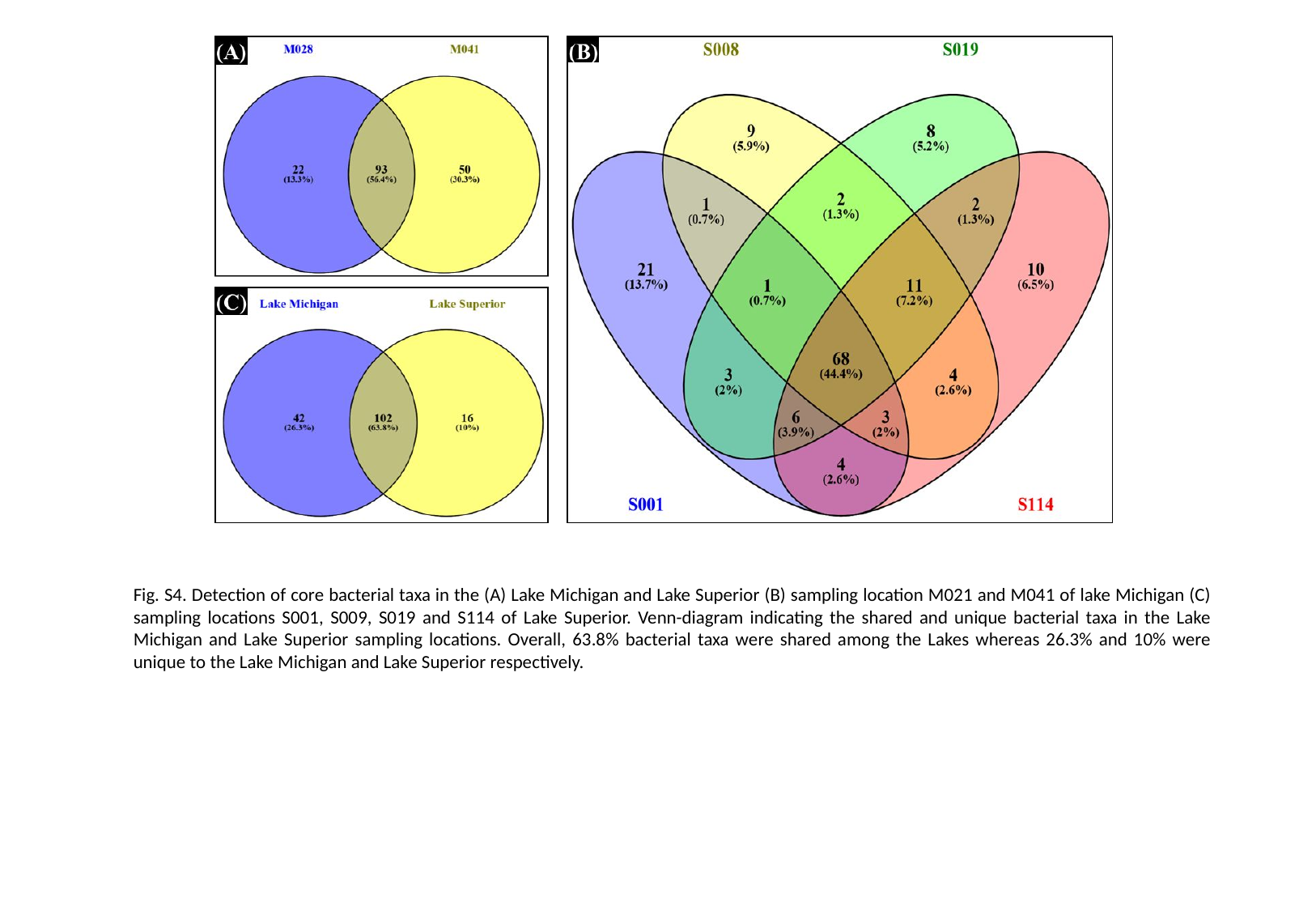

Fig. S4. Detection of core bacterial taxa in the (A) Lake Michigan and Lake Superior (B) sampling location M021 and M041 of lake Michigan (C) sampling locations S001, S009, S019 and S114 of Lake Superior. Venn-diagram indicating the shared and unique bacterial taxa in the Lake Michigan and Lake Superior sampling locations. Overall, 63.8% bacterial taxa were shared among the Lakes whereas 26.3% and 10% were unique to the Lake Michigan and Lake Superior respectively.

## Slide 5
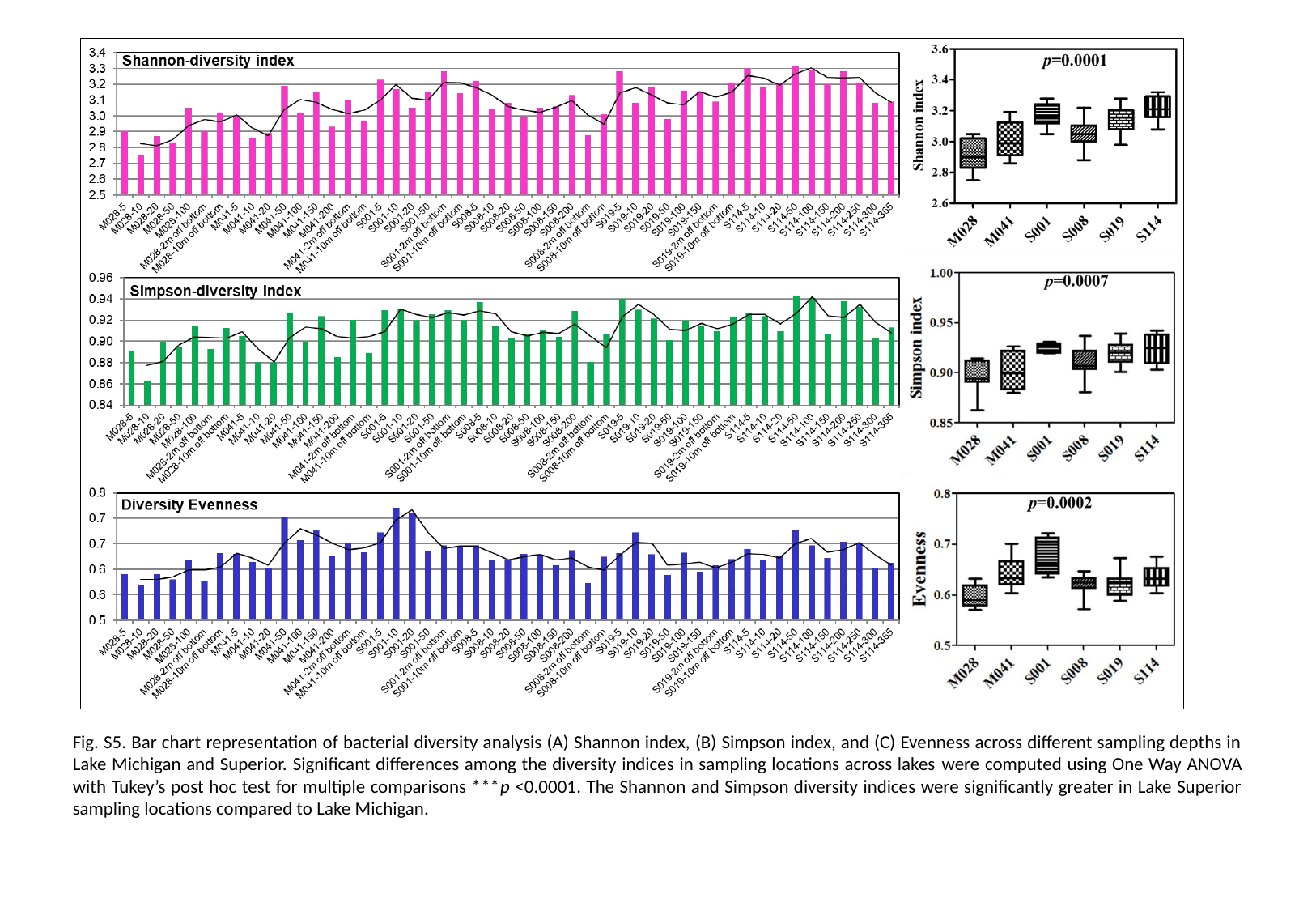

Fig. S5. Bar chart representation of bacterial diversity analysis (A) Shannon index, (B) Simpson index, and (C) Evenness across different sampling depths in Lake Michigan and Superior. Significant differences among the diversity indices in sampling locations across lakes were computed using One Way ANOVA with Tukey’s post hoc test for multiple comparisons ***p <0.0001. The Shannon and Simpson diversity indices were significantly greater in Lake Superior sampling locations compared to Lake Michigan.

## Slide 6
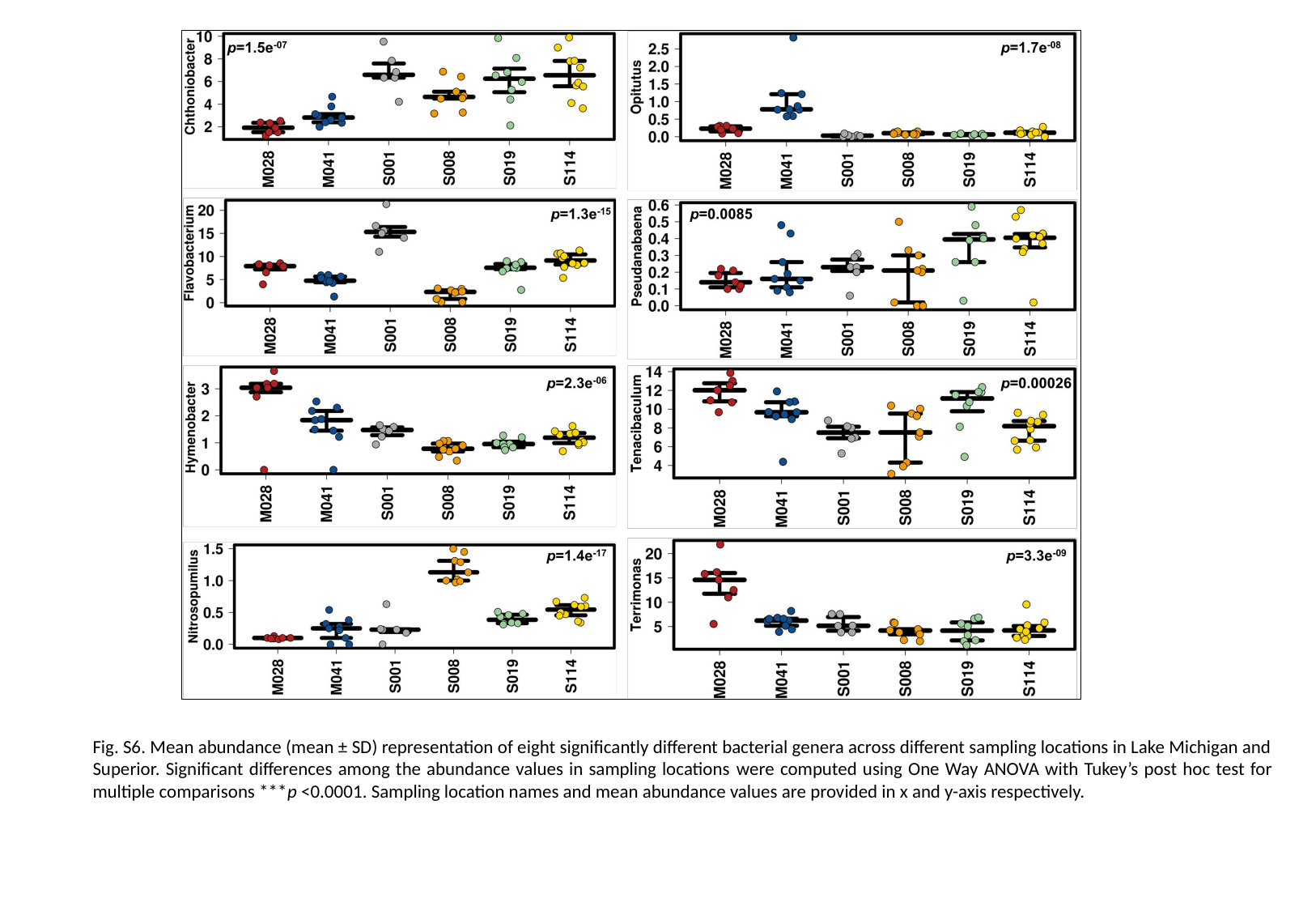

Fig. S6. Mean abundance (mean ± SD) representation of eight significantly different bacterial genera across different sampling locations in Lake Michigan and Superior. Significant differences among the abundance values in sampling locations were computed using One Way ANOVA with Tukey’s post hoc test for multiple comparisons ***p <0.0001. Sampling location names and mean abundance values are provided in x and y-axis respectively.

## Slide 7
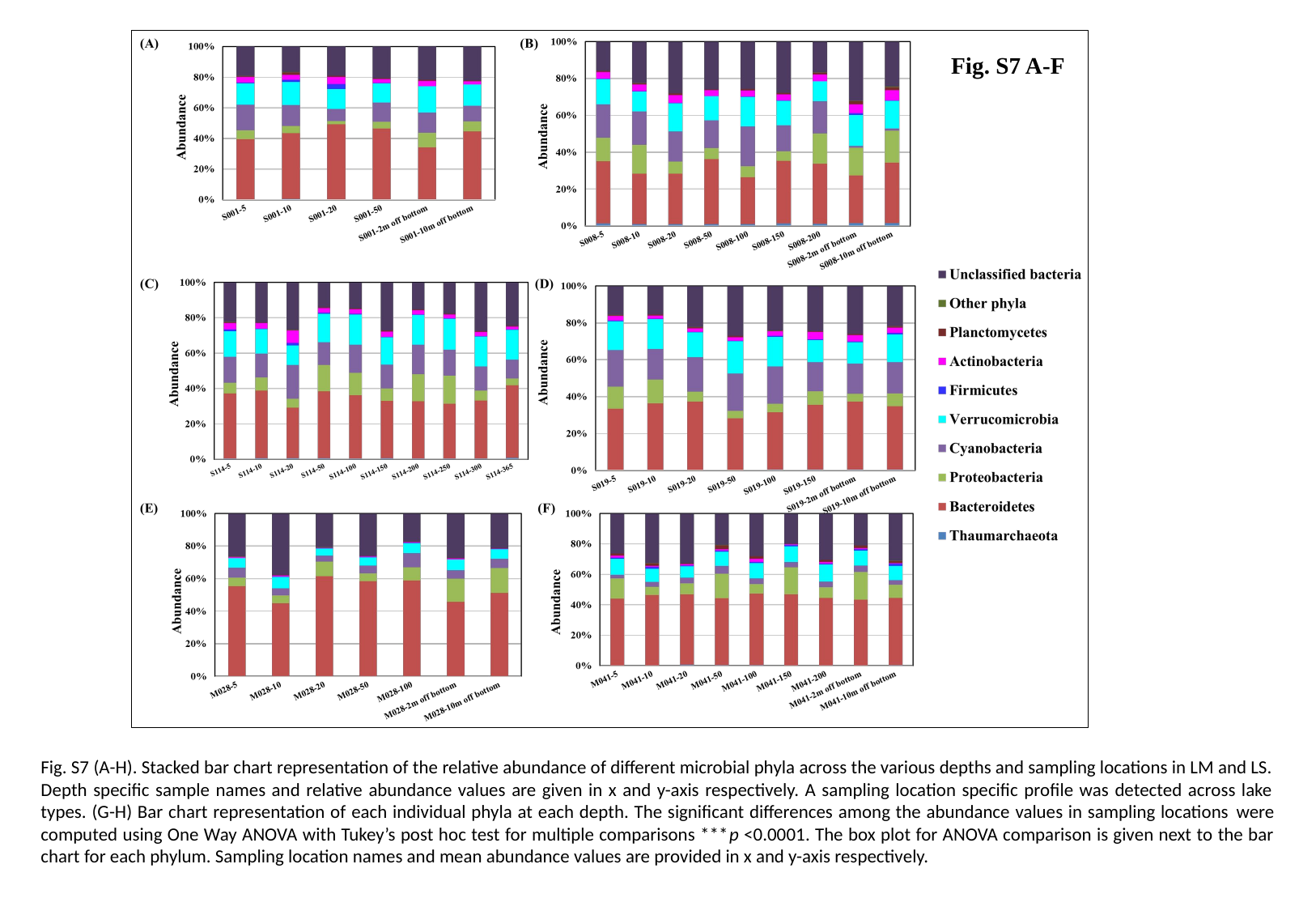

Fig. S7 A-F
Fig. S7 (A-H). Stacked bar chart representation of the relative abundance of different microbial phyla across the various depths and sampling locations in LM and LS. Depth specific sample names and relative abundance values are given in x and y-axis respectively. A sampling location specific profile was detected across lake types. (G-H) Bar chart representation of each individual phyla at each depth. The significant differences among the abundance values in sampling locations were computed using One Way ANOVA with Tukey’s post hoc test for multiple comparisons ***p <0.0001. The box plot for ANOVA comparison is given next to the bar chart for each phylum. Sampling location names and mean abundance values are provided in x and y-axis respectively.

## Slide 8
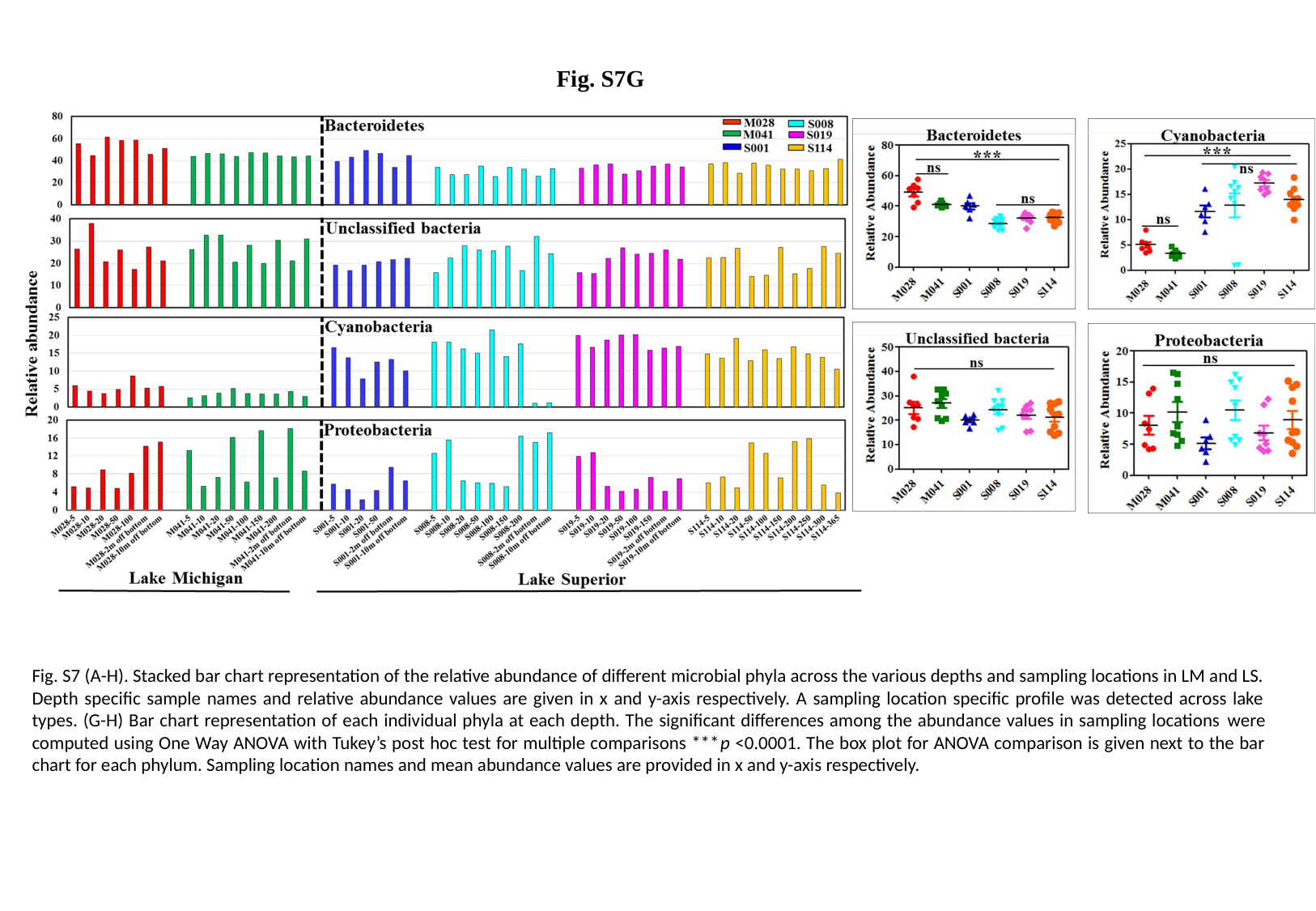

Fig. S7G
Fig. S7 (A-H). Stacked bar chart representation of the relative abundance of different microbial phyla across the various depths and sampling locations in LM and LS. Depth specific sample names and relative abundance values are given in x and y-axis respectively. A sampling location specific profile was detected across lake types. (G-H) Bar chart representation of each individual phyla at each depth. The significant differences among the abundance values in sampling locations were computed using One Way ANOVA with Tukey’s post hoc test for multiple comparisons ***p <0.0001. The box plot for ANOVA comparison is given next to the bar chart for each phylum. Sampling location names and mean abundance values are provided in x and y-axis respectively.

## Slide 9
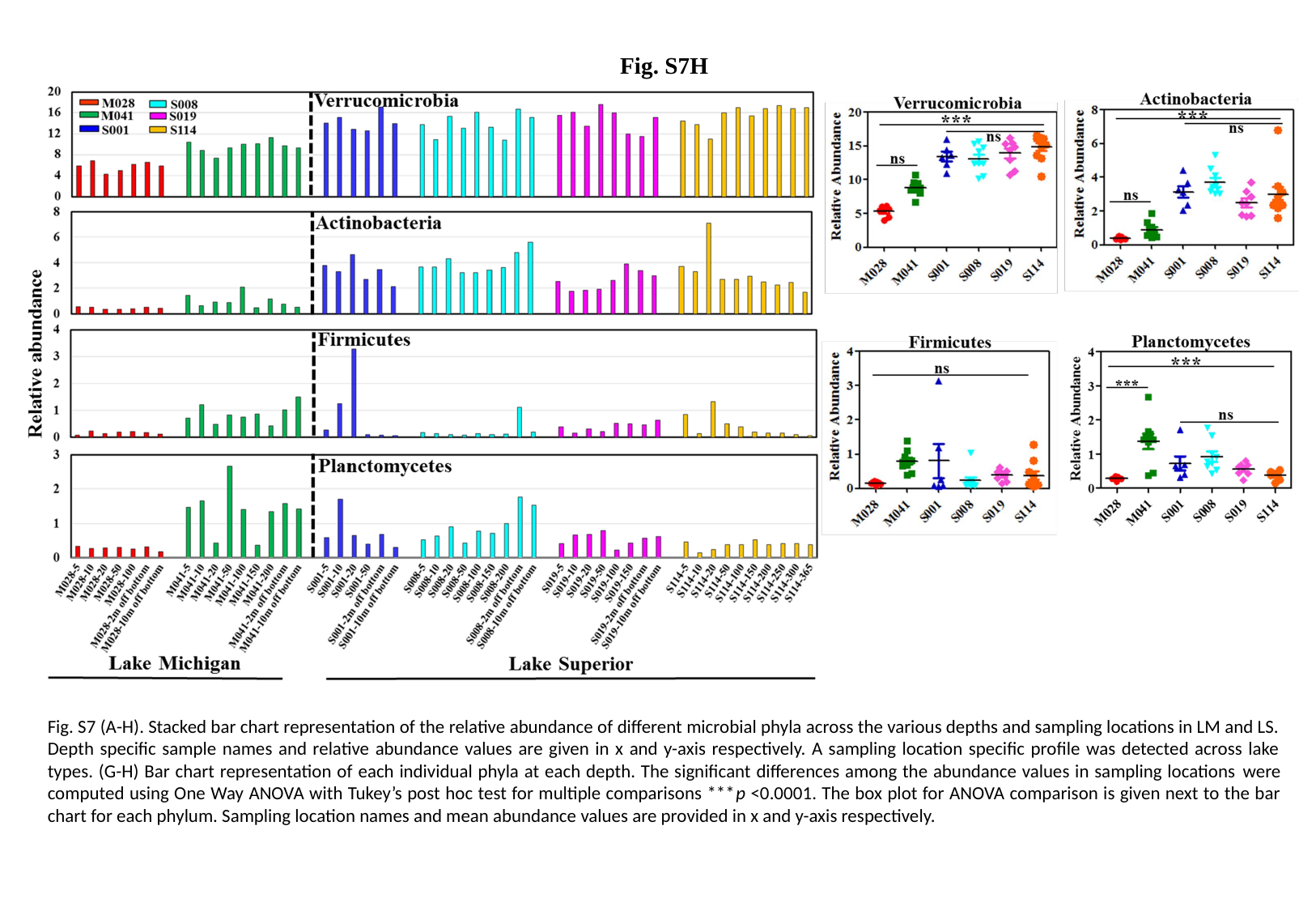

Fig. S7H
Fig. S7 (A-H). Stacked bar chart representation of the relative abundance of different microbial phyla across the various depths and sampling locations in LM and LS. Depth specific sample names and relative abundance values are given in x and y-axis respectively. A sampling location specific profile was detected across lake types. (G-H) Bar chart representation of each individual phyla at each depth. The significant differences among the abundance values in sampling locations were computed using One Way ANOVA with Tukey’s post hoc test for multiple comparisons ***p <0.0001. The box plot for ANOVA comparison is given next to the bar chart for each phylum. Sampling location names and mean abundance values are provided in x and y-axis respectively.

## Slide 10
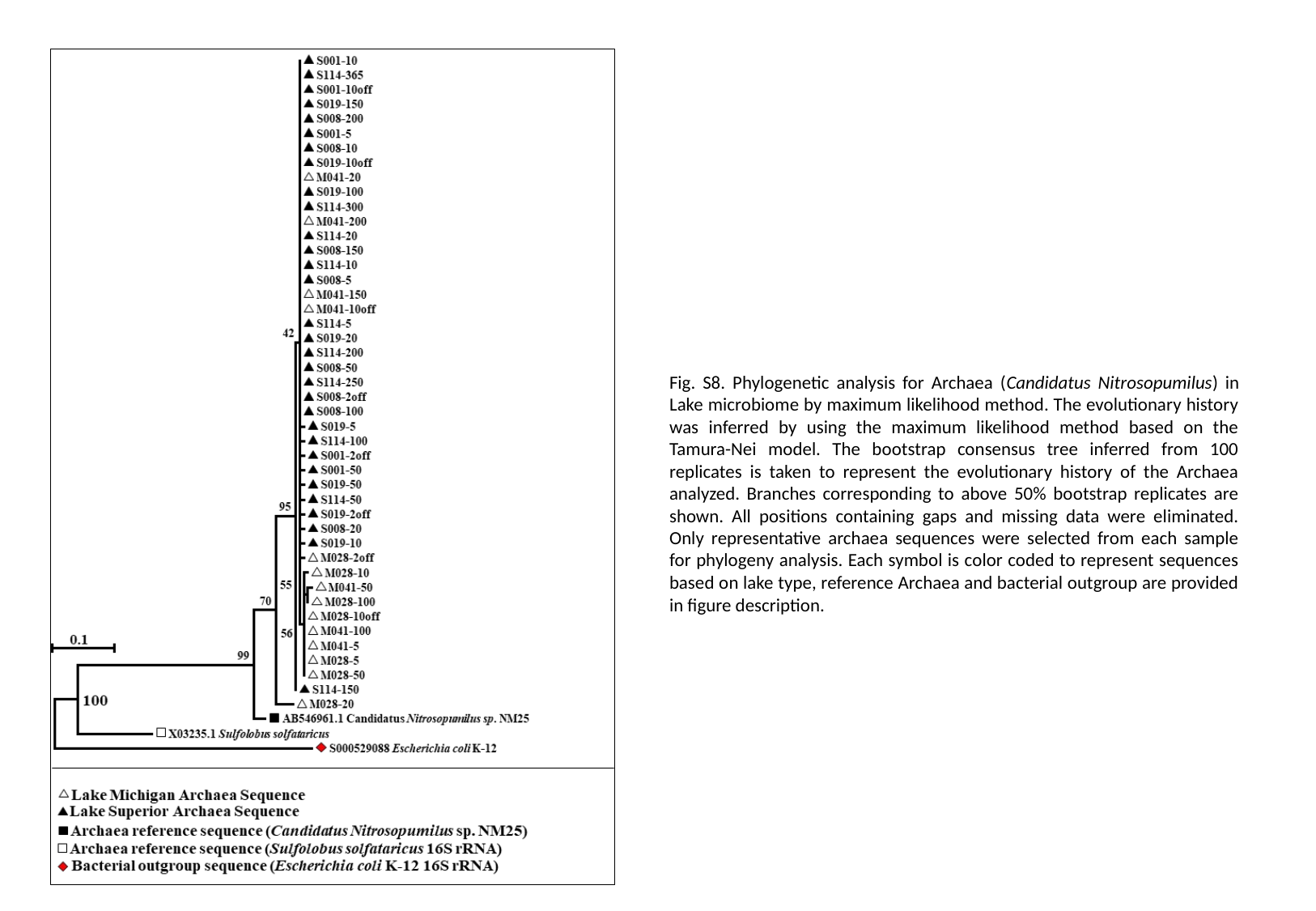

Fig. S8. Phylogenetic analysis for Archaea (Candidatus Nitrosopumilus) in Lake microbiome by maximum likelihood method. The evolutionary history was inferred by using the maximum likelihood method based on the Tamura-Nei model. The bootstrap consensus tree inferred from 100 replicates is taken to represent the evolutionary history of the Archaea analyzed. Branches corresponding to above 50% bootstrap replicates are shown. All positions containing gaps and missing data were eliminated. Only representative archaea sequences were selected from each sample for phylogeny analysis. Each symbol is color coded to represent sequences based on lake type, reference Archaea and bacterial outgroup are provided in figure description.

## Slide 11
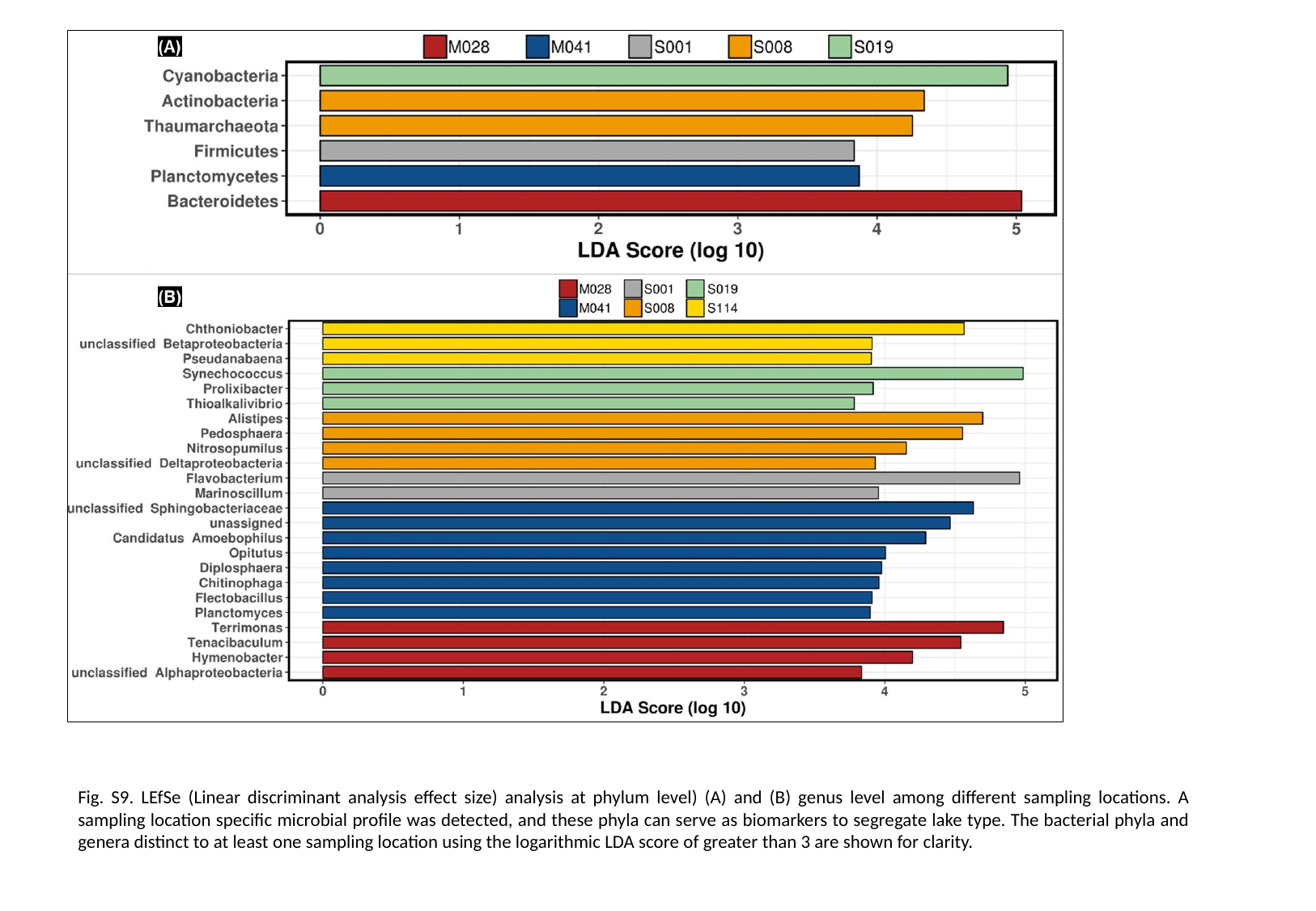

Fig. S9. LEfSe (Linear discriminant analysis effect size) analysis at phylum level) (A) and (B) genus level among different sampling locations. A sampling location specific microbial profile was detected, and these phyla can serve as biomarkers to segregate lake type. The bacterial phyla and genera distinct to at least one sampling location using the logarithmic LDA score of greater than 3 are shown for clarity.

## Slide 12
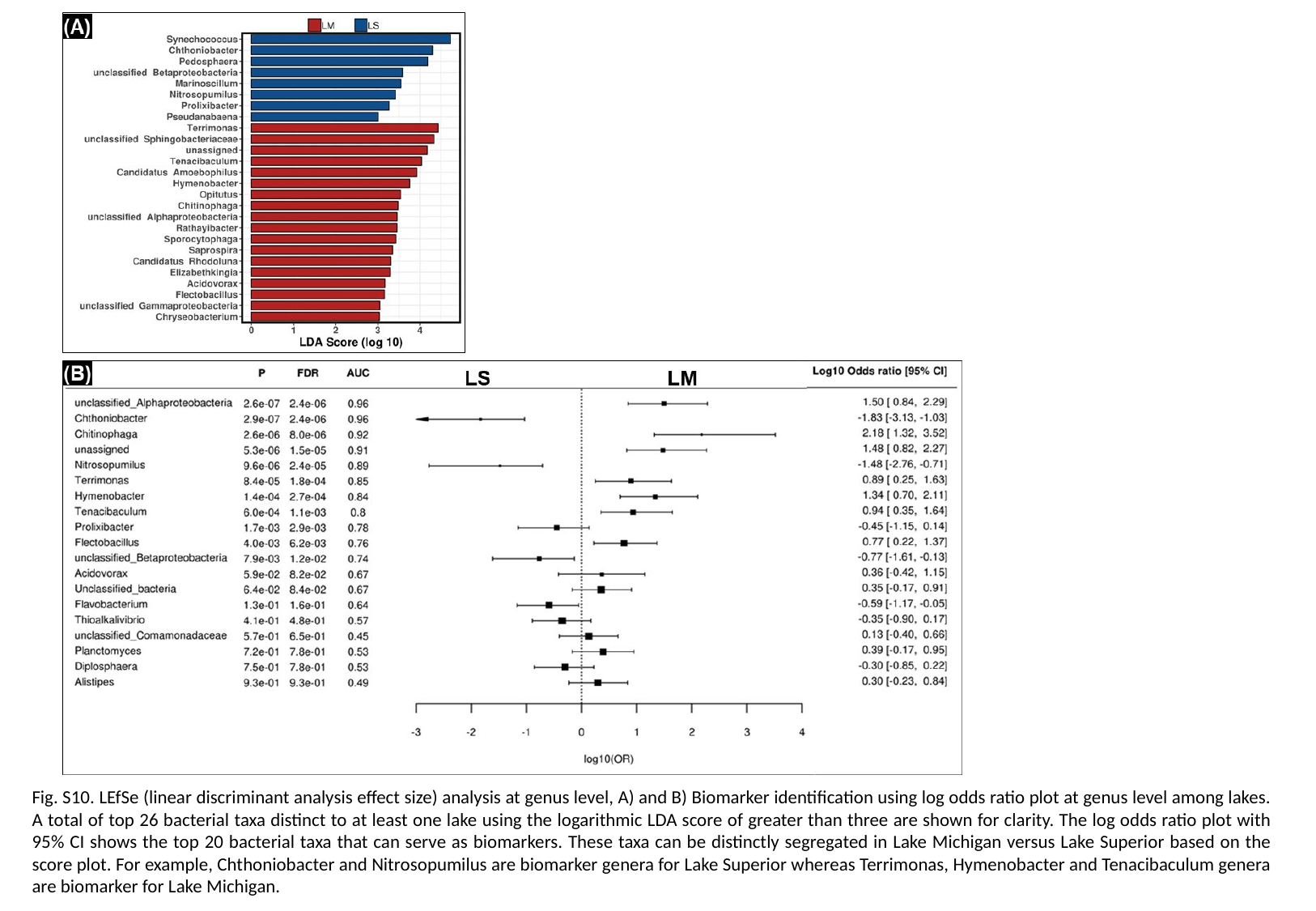

Fig. S10. LEfSe (linear discriminant analysis effect size) analysis at genus level, A) and B) Biomarker identification using log odds ratio plot at genus level among lakes. A total of top 26 bacterial taxa distinct to at least one lake using the logarithmic LDA score of greater than three are shown for clarity. The log odds ratio plot with 95% CI shows the top 20 bacterial taxa that can serve as biomarkers. These taxa can be distinctly segregated in Lake Michigan versus Lake Superior based on the score plot. For example, Chthoniobacter and Nitrosopumilus are biomarker genera for Lake Superior whereas Terrimonas, Hymenobacter and Tenacibaculum genera are biomarker for Lake Michigan.

## Slide 13
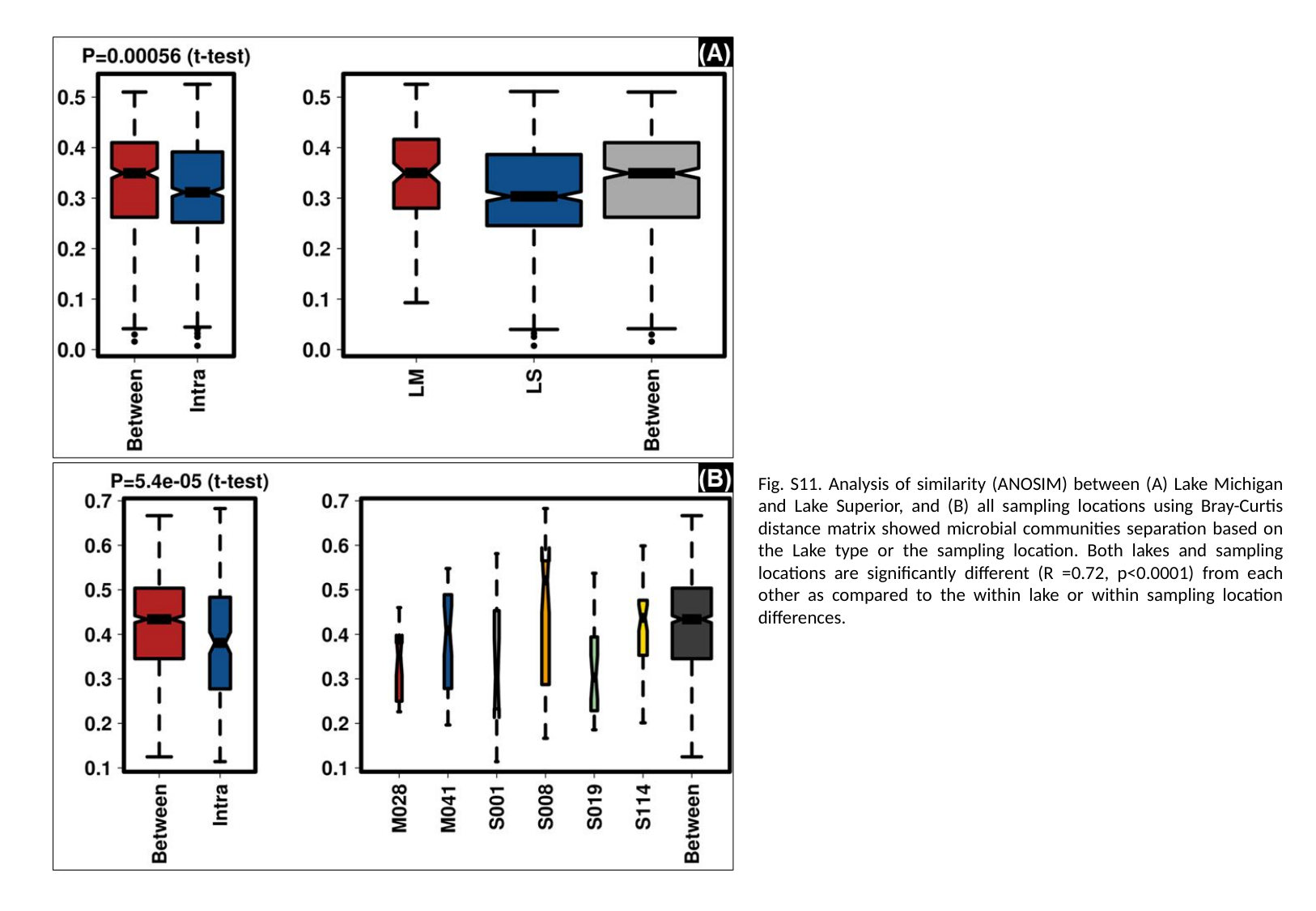

Fig. S11. Analysis of similarity (ANOSIM) between (A) Lake Michigan and Lake Superior, and (B) all sampling locations using Bray-Curtis distance matrix showed microbial communities separation based on the Lake type or the sampling location. Both lakes and sampling locations are significantly different (R =0.72, p<0.0001) from each other as compared to the within lake or within sampling location differences.

## Slide 14
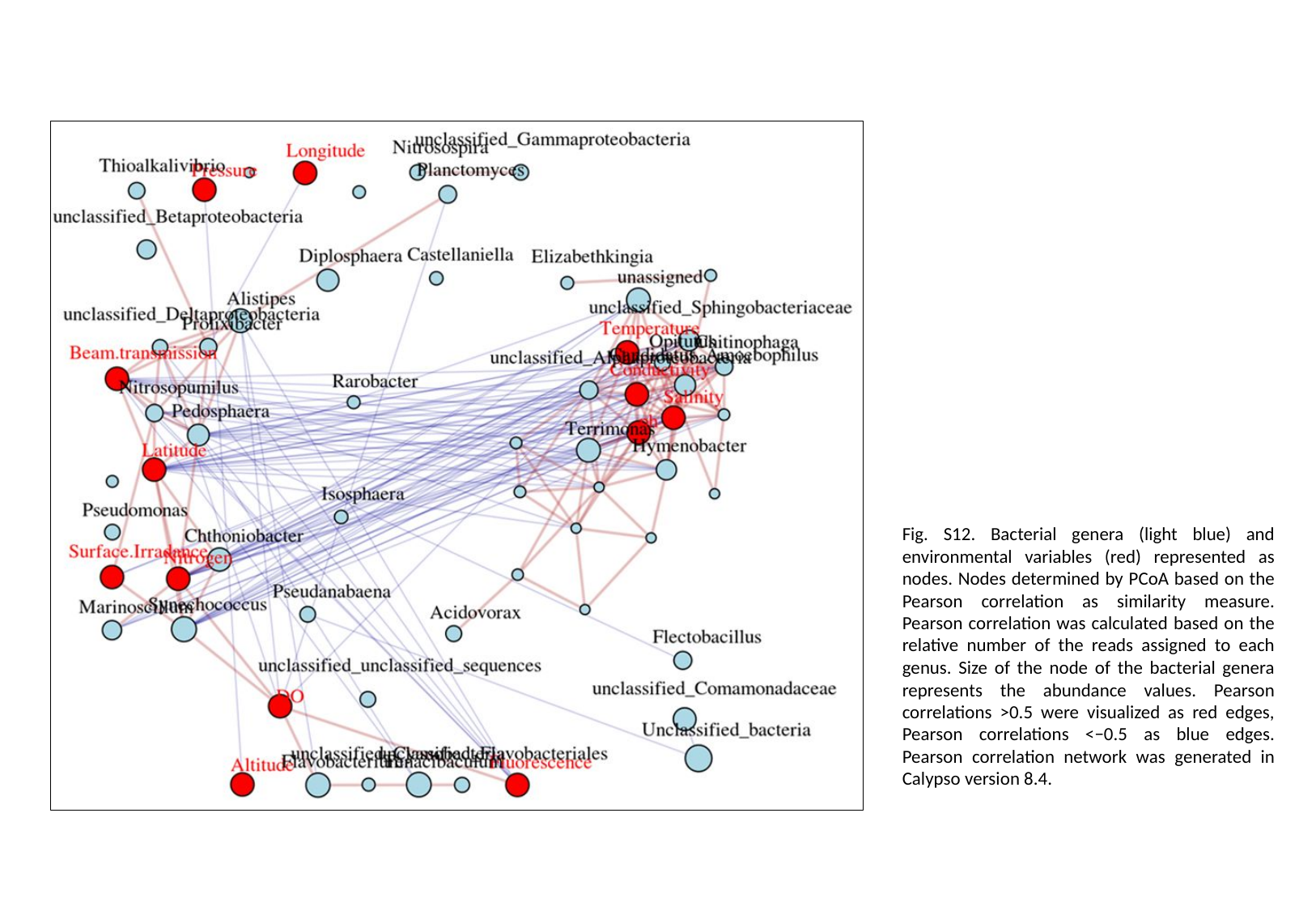

Fig. S12. Bacterial genera (light blue) and environmental variables (red) represented as nodes. Nodes determined by PCoA based on the Pearson correlation as similarity measure. Pearson correlation was calculated based on the relative number of the reads assigned to each genus. Size of the node of the bacterial genera represents the abundance values. Pearson correlations >0.5 were visualized as red edges, Pearson correlations <−0.5 as blue edges. Pearson correlation network was generated in Calypso version 8.4.

## Slide 15
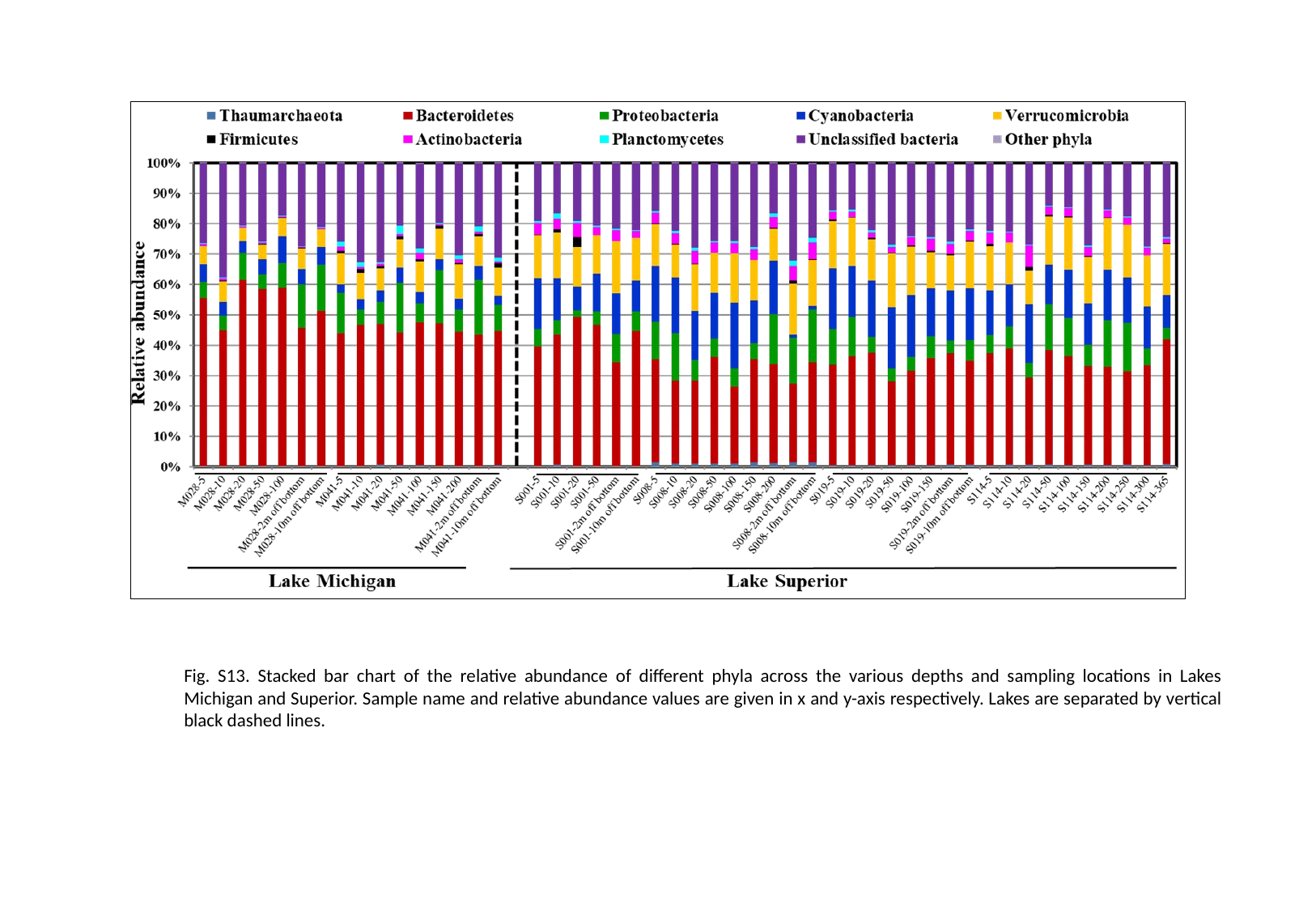

Fig. S13. Stacked bar chart of the relative abundance of different phyla across the various depths and sampling locations in Lakes Michigan and Superior. Sample name and relative abundance values are given in x and y-axis respectively. Lakes are separated by vertical black dashed lines.

## Slide 16
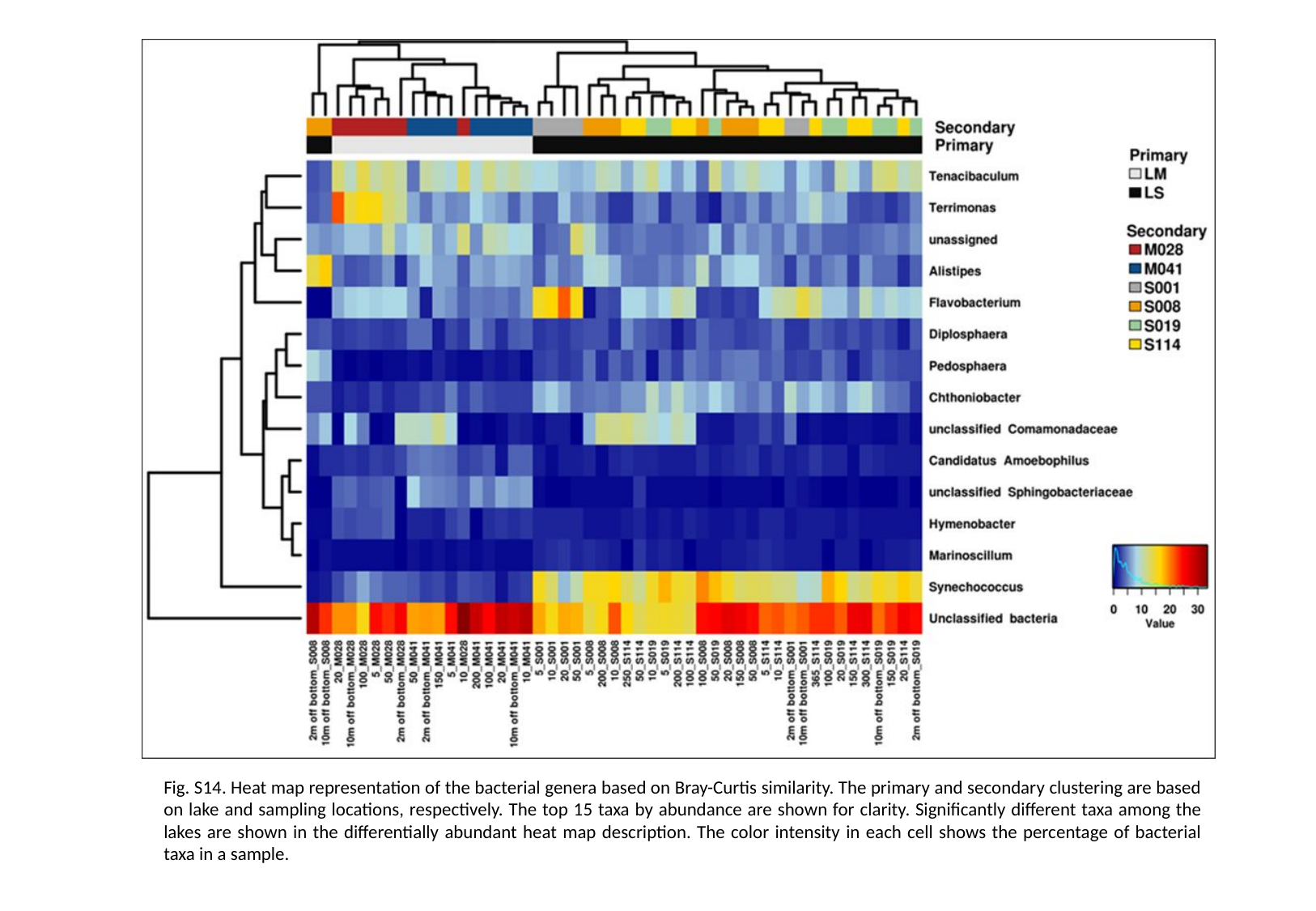

Fig. S14. Heat map representation of the bacterial genera based on Bray-Curtis similarity. The primary and secondary clustering are based on lake and sampling locations, respectively. The top 15 taxa by abundance are shown for clarity. Significantly different taxa among the lakes are shown in the differentially abundant heat map description. The color intensity in each cell shows the percentage of bacterial taxa in a sample.
